# Metal–Organic Frameworks as Sensors for Human Amyloid Diseases

**DOI:** 10.1021/acssensors.2c02741

**Published:** 2023-03-09

**Authors:** José P. Leite, Flávio Figueira, Ricardo F. Mendes, Filipe A. Almeida Paz, Luís Gales

**Affiliations:** † i3S−Instituto de Investigação e Inovação em Saúde, Rua Alfredo Allen, 208, 4200-135 Porto, Portugal; ‡ IBMC−Instituto de Biologia Molecular e Celular Universidade do Porto, Rua Alfredo Allen, 208, 4200-135 Porto, Portugal; § Programa Doutoral em Biologia Molecular e Celular (MCbiology), ICBAS−Instituto de Ciências Biomédicas Abel Salazar, Rua de Jorge Viterbo Ferreira 228, 4050-313 Porto, Portugal; ∥ Department of Chemistry, CICECO−Aveiro Institute of Materials, University of Aveiro, 3810-193 Aveiro, Portugal; ⊥ ICBAS−Instituto de Ciências Biomédicas Abel Salazar, Rua de Jorge Viterbo Ferreira 228, 4050-313 Porto, Portugal

**Keywords:** metal−organic frameworks, amyloid diseases, biosensor, amyloid inhibition, amyloid biomarker, Alzheimer’s disease, diagnostic, immunosensor

## Abstract

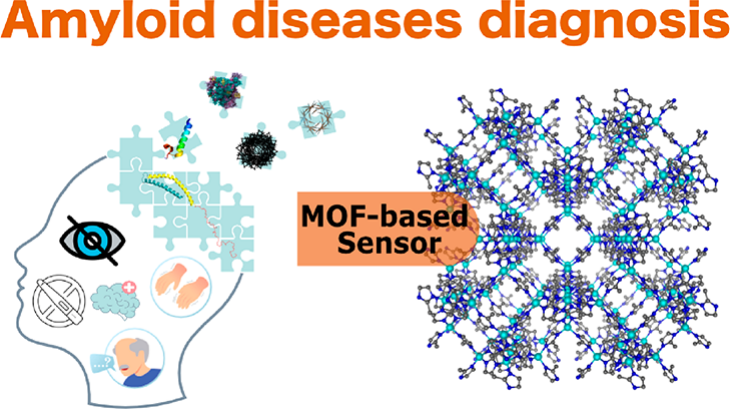

Metal–organic frameworks (MOFs) are versatile compounds with emergent applications in the fabrication of biosensors for amyloid diseases. They hold great potential in biospecimen protection and unprecedented probing capabilities for optical and redox receptors. In this Review, we summarize the main methodologies employed in the fabrication of MOF-based sensors for amyloid diseases and collect all available data in the literature related to their performance (detection range, limit of detection, recovery, time of analysis, among other parameters). Nowadays, MOF sensors have evolved to a point where they can, in some cases, outperform technologies employed in the detection of several amyloid biomarkers (amyloid β peptide, α-synuclein, insulin, procalcitonin, and prolactin) present in biological fluids, such as cerebrospinal fluid and blood. A special emphasis has been given by researchers on Alzheimer’s disease monitoring to the detriment of other amyloidosis that are underexploited despite their societal relevance (e.g., Parkinson’s disease). There are still important obstacles to overcome in order to selectively detect the various peptide isoforms and soluble amyloid species associated with Alzheimer’s disease. Furthermore, MOF contrast agents for imaging peptide soluble oligomers in living humans are also scarce (if not nonexistent), and action in this direction is unquestionably required to clarify the contentious link between the amyloidogenic species and the disease, guiding research toward the most promising therapeutic strategies.

## Amyloid Biomarkers

**Figure 1:**
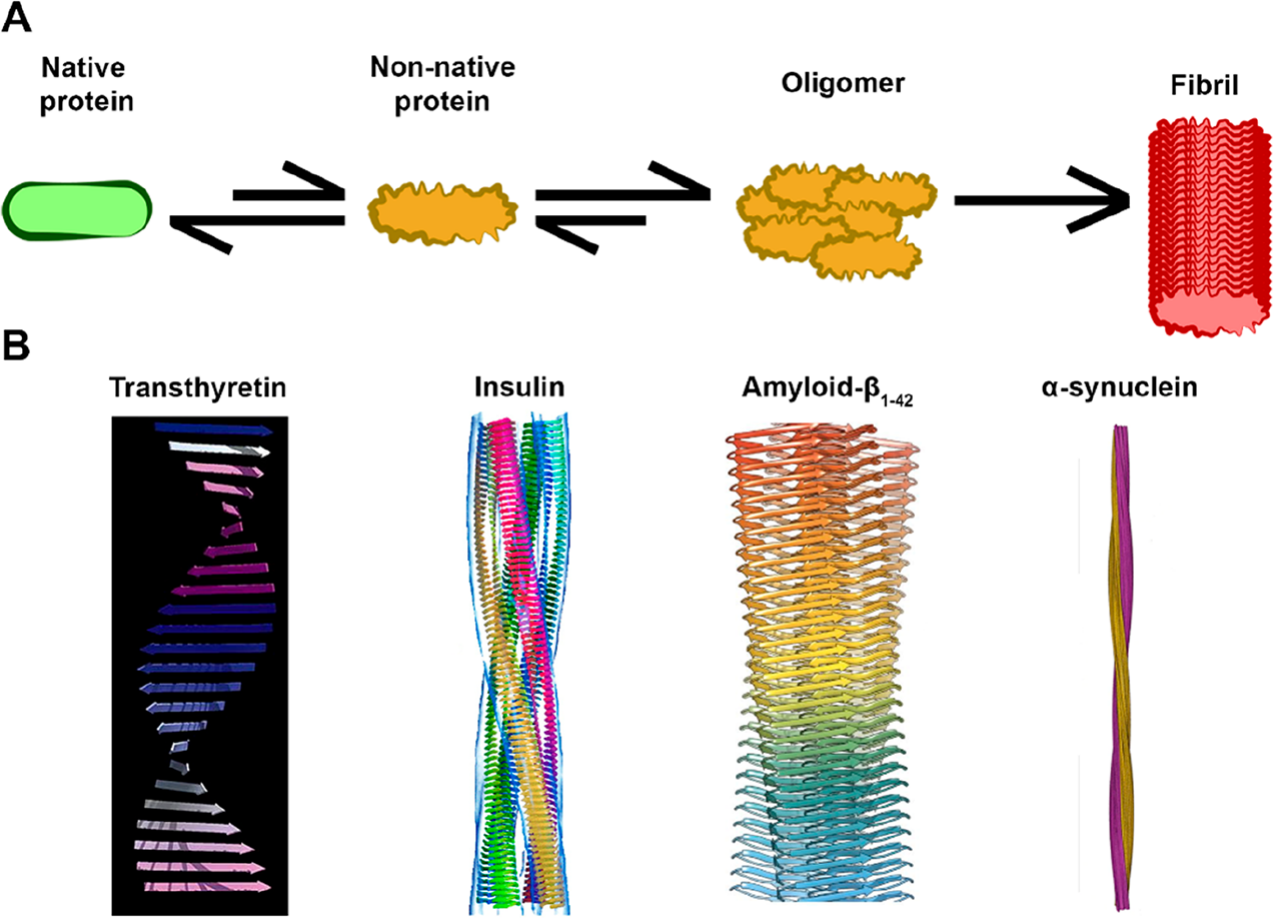
(A) Schematic representation of an amyloid formation pathway. The catalyst of this process might be denaturation, overexpression, or cleavage of a normally folded protein, as well as production of an intrinsically disordered protein.^2^ Figure prepared with icons sourced from ref (3). (B) Examples of structural models of amyloid protofilaments of different origins (transthyretin, insulin, amyloid-β peptide, and α-synuclein), exhibiting the characteristic β-strand composition. (4). Copyright 1996 Elsevier. Adapted with permission from ref (4).

A key lesson that emerges from the management and treatment of amyloid diseases is that early diagnosis is essential to lessen disease progression. Often, several years or decades before pathophysiological changes are denoted, an increase in protein concentration (or an aberrant form of a protein) in a body fluid begins.^1^ Robust assays to lower the detection threshold of these biomarkers can be designed based on the same concepts, because the mechanism of amyloid fibril formation associated with most amyloidosis shares common features at the molecular level (Figure 1).

Currently, there are around 20 known human amyloid diseases.^5^ Deposition of protein aggregates associated with these diseases can occur at localized tissues (e.g., localized insulin-derived amyloidosis or medullary thyroid carcinoma), associated with the central nervous system (e.g., Alzheimer’s and Parkinson’s diseases), or systemic (e.g., apolipoprotein amyloidosis or hereditary non-neuropathic systemic amyloidosis) with a spontaneous or hereditary nature. According to the World Health Organization, 60–70% of the currently diagnosed dementia cases worldwide are Alzheimer’s disease patients.^6^ MOF-based (MOF, metal–organic framework) sensors have been mostly designed to address Alzheimer’s disease monitoring, with a few directed toward Parkinson’s disease and other amyloid diseases.

### Alzheimer’s Disease Biomarkers

The first striking symptom of Alzheimer’s disease (AD) is a progressive loss of short-term memory.^7^ As the disease advances, more debilitating symptoms appear, such as loss of orientation, language impairment, decision-making hindrance, and lack of judgment.^7,8^ Medically it is diagnosed by the histological detection of two pathological features in brain tissue: intracellular tau protein deposition of neurofibrillary tangles (NFTs) and extracellular deposition of amyloid-β (Aβ) peptide plaques.^9^ The discovery in 1984 of Aβ as the building block of extracellular amyloid plaques strongly supported the amyloid cascade hypothesis.^10^ Aβ peptides are typically 36–43 residues in length, with Aβ_1–40_ and Aβ_1–42_ (subscript numbers indicate amino acid length; note that the term Aβ will be generically used when the authors do not disclose the precise peptide used). The detection of Aβ levels is crucially carried out by the invasive collection of cerebrospinal fluid (CSF), where concentrations higher than 0.1 nM may indicate nonbenign accumulation,^11^ while serum concentrations above 36 pM are associated with mild dementia.^12^ Other techniques in monitoring Aβ levels in living humans include PET (positron emission tomography) with Pittsburgh compound B and magnetic resonance imaging (MRI) for the detection of fibrillar deposits in the brain.^1,9^

The amyloid hypothesis was, however, never consensual and is being threatened nowadays. Several anti-Aβ therapeutic pipelines have recently failed the clinical trials with the exception of the controversial FDA approval of aducanumab,^13^ a monoclonal antibody that removes the Aβ plaques.^14^ Despite the uncertain role played by Aβ in Alzheimer’s disease, the plasma Aβ_1–42_/Aβ_1–40_ ratio seems to be a good correlation with the presence of amyloid aggregates in the brain.^15^

Other biomarkers have been correlated with AD. For example, a decrease in acetylcholine synthesis, a neurotransmitter associated with brain functions such as learning and memory, has been linked to Alzheimer’s onset.^16^ Based on these findings, four out of the only five approved clinical drugs for AD are cholinesterase inhibitors.^17^ In addition, missense mutations of PSEN1 (a component of the γ-secretase complex involved in Aβ synthesis), such as the well-documented E280A, lead to the preferential formation of longer, more aggregation-prone Aβ forms (such as Aβ_1–42_ and Aβ_1–43_).^18^

### Parkinson’s Disease Biomarkers

Next to Alzheimer’s, Parkinson’s disease (PD) is arguably the second most recognized neurodegenerative disease, with around 10 million diagnosed patients worldwide. Symptoms include bradykinesia, tremors while resting, and dementia.^19^ The disease is caused by degeneration of dopaminergic neurons in the *substantia nigra* of the brain. The striking histological feature for Parkinson’s is amyloid aggregates of the nuclear synaptic protein α-synuclein, constituting the characteristic Lewy bodies or Lewy neurites.^19^ α-Synuclein is an intrinsically disordered protein that, under particular conditions, self-assembles into oligomers and, ultimately, mature fibrils. The oligomers are thought to exert neurotoxic effects through the generation of reactive oxygen species and permeabilization of vesicles carrying the neurotransmitter dopamine.^19^ When clinical symptoms are present, around 50% of dopaminergic neurons are already irremediably lost. It is thus of critical importance to develop strategies for early diagnosis. Naturally, sensitive and specific detection of soluble α-synuclein oligomer, that typically occurs in the pg mL^–1^ range in circulating fluids, is of key importance.^20^

### Other Amyloid Biomarkers

Many other peptides or proteins can form disease-associated amyloid deposits, being relevant for preventing amyloidosis. Insulin is a peptide hormone produced by the pancreatic β-cells and is responsible for cellular glucose uptake.^21^ Despite being a rare occurrence, insulin may aggregate into amyloid fibrils at the injection site (for example, lower abdomen) and cause localized insulin-derived amyloidosis (LIDA).^22^ The subcutaneous amyloid aggregates are often mistaken for tumor growth or the more common insulin-lipohypertrophy, which explains why LIDA is thought to be an underdiagnosed diabetes-related complication.^22^ LIDA leads to dysregulation of glycemic bloodstream control and may even result in necrosis of the tissue surrounding the amyloid deposit.^23^

Other human amyloidosis caused by polypeptide hormones is associated with endocrine tumors, such as medullary thyroid cancer (MTC, one of the most aggressive forms of thyroid cancer). A hallmark of MTC is the occurrence of amyloid deposits, the main building block of which is calcitonin. Two main aspects hinder however the widespread use of calcitonin for MTC diagnosis.^24^ First, the low incidence of this carcinoma makes it less competitive to implement current testing methods, mainly due to its high costs.^25^ Second, calcitonin has a low half-life, which is easily degraded in the serum by proteases. As an alternative, procalcitonin is much more stable *in vivo*, and its levels correlate well with those for calcitonin.^26^ In this context, the development of highly sensitive and cost-effective sensors for procalcitonin serum concentrations may help the rapid diagnosis of amyloid formation in the context of MTC.

Prolactin constitutes yet another example of a hormone that can trigger amyloid formation in an endocrine tumor. It is secreted by the pituitary gland (known as hypophysis) and presents one of the most diverse functional roles among the known hormones. In the case of pituitary prolactinoma, amyloid formation can occur by prolactin deposition.^27^ This form of tumor leads to prolactin overexpression, and despite being noncancerous, it can cause problems such as infertility or vision impairment. Pharmacological or surgical removals are the current therapeutic options. Circulating prolactin levels above 0.8 nM may indicate pituitary prolactinoma.

Point mutations in lysozyme and apolipoprotein IV may cause autosomal dominant hereditary systemic amyloidosis. The amyloids may then deposit in various organs, such as the liver, kidneys, heart, and digestive tract. For the lysozyme-caused hereditary non-neuropathic systemic amyloidosis, amyloid accumulation typically reveals to be fatal around the fifth decade of life of the patient,^28^ while for ApoA4 amyloidosis deposits are typically confined to certain tissues, such as the renal medulla or cardiac tissue, and are usually not life-threatening.^29^ An accurate (early) diagnosis is critical to avoid employing unnecessarily aggressive therapy (e.g., chemotherapy, or organ/stem cell transplants).^30^

## MOFs in Amyloid Diseases Diagnostic

The development of ultrasensitive and highly selective sensors is significant for early diagnosis and monitoring of amyloid diseases, and is critical for aneffective treatment. Despite many efforts to develop tools to detect, monitor, and manipulate amyloidosis biomarkers in biological samples, the low concentrations of these species and cross reactivity between monomers and oligomers hamper the development of highly sensitive and reliable detection techniques.^31^ To enhance the sensing performance, such as sensitivity, selectivity, and response speed for diagnostic biomarkers of amyloid diseases, researchers have exploited until now a wide variety of carbon-based nanomaterials, conductive polymers, quantum dots, noble metals, and MOFs.^32^

MOFs in general have received increased attention on account of their potential application in a wide variety of fields, such as adsorption, environment, storage, separation, and sensing.^33^ Research on their biomedical applications has gained traction in the past decade,^34^ mostly resulting from their astonishing structural properties,^35^ which include permanent porosity, exceptional specific surface areas, tailorable pore size/structure, versatile modifications, and biocompatibility.^36^ By virtue of these chemical and physical attributes, they have attracted tremendous interest as sensitive platforms for anchoring diverse probes (e.g., antibodies, DNA, or aptamers) for the construction of biosensors.^37^ The integration of biomacromolecules within MOFs is typically achieved following mostly three simple strategies involving bioconjugation (outer surface covalent attachment or adsorption induced by the electrostatic interactions to the MOF), infiltration inside the pores via diffusion processes, and encapsulation during MOF synthesis.^38^

Design of MOFs toward the inclusion of specific guest molecules can lead to modifications in the optical, electrical, photophysical, or mechanical properties of the whole framework.^39^ These properties embody MOFs with a diverse array of applications as biosensing platforms aimed at fast diagnosis of illnesses like cancer or diabetes, detection of pathogens, quantification of drugs and their metabolites, and detection of analytes in biological samples, with the concomitant disease diagnosis through rapid tests.^40^ These materials serve as outstanding supports for the incorporation of biomolecules combining the properties of both constituents, creating stable microenvironments for the protection of biomolecules, conferring increased stability to the sensing motif and ease of use of the sensor without the need for refrigeration or complex laboratory setup protection.^41^ Extensive overviews of MOFs employed as biosensors and other biomedical and industrial applications are available in the literature.^4d,6,42^ Recent *in vivo* studies on the toxicity of MOFs show that these are, in most cases, nontoxic.^34a^ The preparation of MOFs with biocompatible metals and ligands at the nanoscale circumvents some stability issues when immersed for prolonged times in certain physiological conditions. In particular, those composed by saline buffers (e.g., phosphate saline buffer) where MOFs such ZIF-8, MIL-101, and UiO-66 have been demonstrated to be unstable.^43^ This behavior contrasts with *in vivo* studies where MOFs from the MIL family were shown to be stable under different biological media, allowing their use in a wide range of clinical fields such as contrast agents for medical imaging.^44^

**Figure 2:**
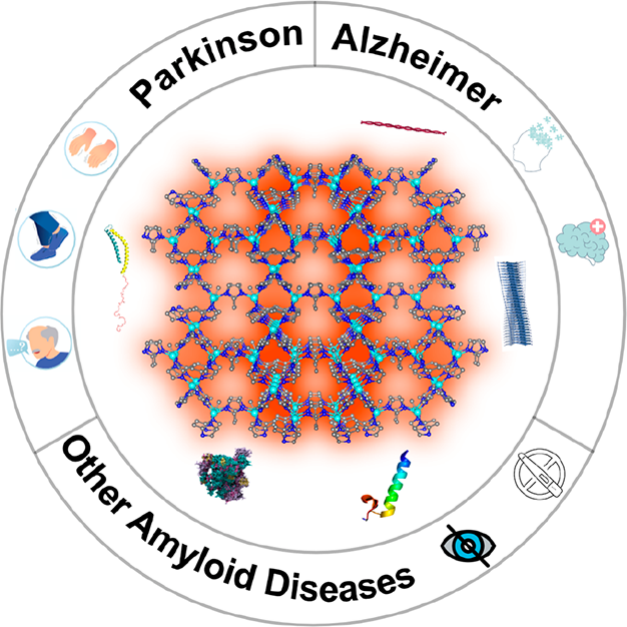
MOF sensors for amyloid disease diagnostics.

In this Review, we explore the usage of MOFs in a particular area of biomedicine: human amyloid diseases. While presenting an overview of recent works, pinpointing remarkable results in different biomedical areas, we further focus on MOFs used as supports or even active components for amyloid diagnostic sensors (Figure 2).

**Figure 3:**
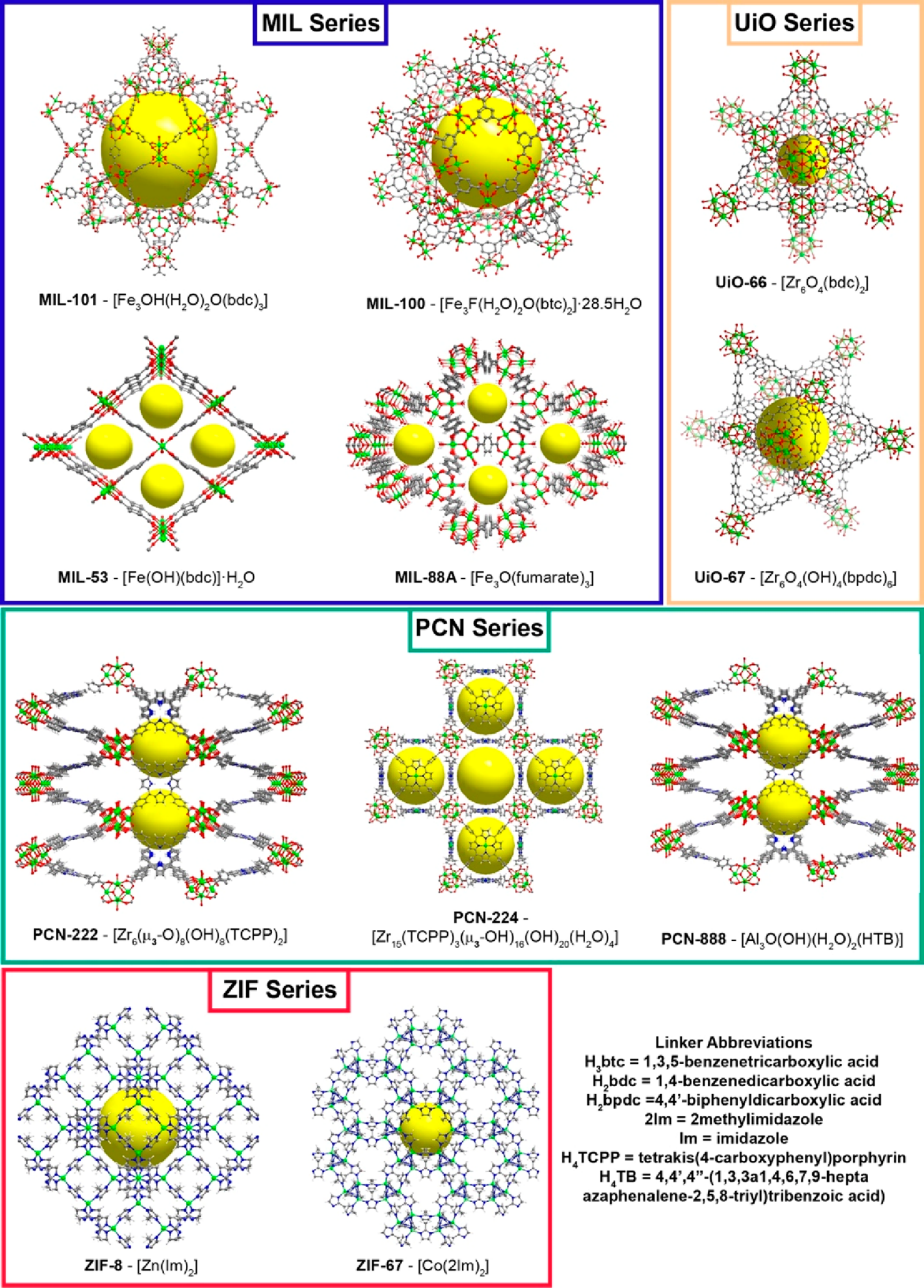
Structural representation of the most common families of MOFs presented in this Review. The yellow sphere represents the pore/cavity present in each material. Legend: green, metal center (Fe, Zn, Co or Zr); gray, carbon; blue, nitrogen; light gray, hydrogen.

It is not by accident that the most employed networks are those of the MIL series, namely, MIL-100 ([Fe_3_F(H_2_O)_2_O(btc)_2_]·28.5H_2_O), MIL-101 ([Fe_3_OH(H_2_O)_2_O(bdc)_3_]), MIL-53 ([Fe(OH)(bdc)]·H_2_O), and MIL-88A ([Fe_3_O(fumarate)_3_]) (where H_3_btc = 1,3,5-benzenetricarboxylic acid and H_2_bdc = 1,4-benzenedicarboxylic acid), bearing iron metal nodes within their structures that ensure a good biocompatibility and a good *in vivo* elimination (Figure 3).^45^ The same can be expected for the ZIF family, namely, ZIF-67 ([Co(2Im)_2_]) and ZIF-8 ([Zn(Im)_2_]) (where 2Im = 2-methylimidazole and Im = imidazole).

Taking advantage of their stability in a wide range of pHs, zirconium-based MOFs, specifically those forming the UiO series such as UiO-66 ([Zr_6_O_4_(bdc)_2_]) and UiO-67 ([Zr_6_O_4_(OH)_4_(bpdc)_6_], where H_2_bpdc = 4,4′-biphenyldicarboxylic acid) were studied (Figure 3).^46^ In recent years PCN materials such as PCN-222 ([Zr_6_(μ_3_-O)_8_(OH)_8_(TCPP)_2_], PCN-224 ([Zr_15_(TCPP)_3_(μ_3_–OH)_16_(OH)_20_(H_2_O)_4_]), and PCN-888 ([Al_3_O(OH)(H_2_O)_2_(HTB)]) (where H_4_TCPP = tetrakis(4-carboxyphenyl)porphyrin and H_4_TB = 4,4′,4″-(1,3,3a1,4,6,7,9-heptaazaphenalene-2,5,8-triyl)tribenzoic acid) have been explored because of their rich optical profiles (absorption and emission) and their capability to generate singlet oxygen giving rise to multifunctional materials (Figure 3).^47^

**Figure 4:**
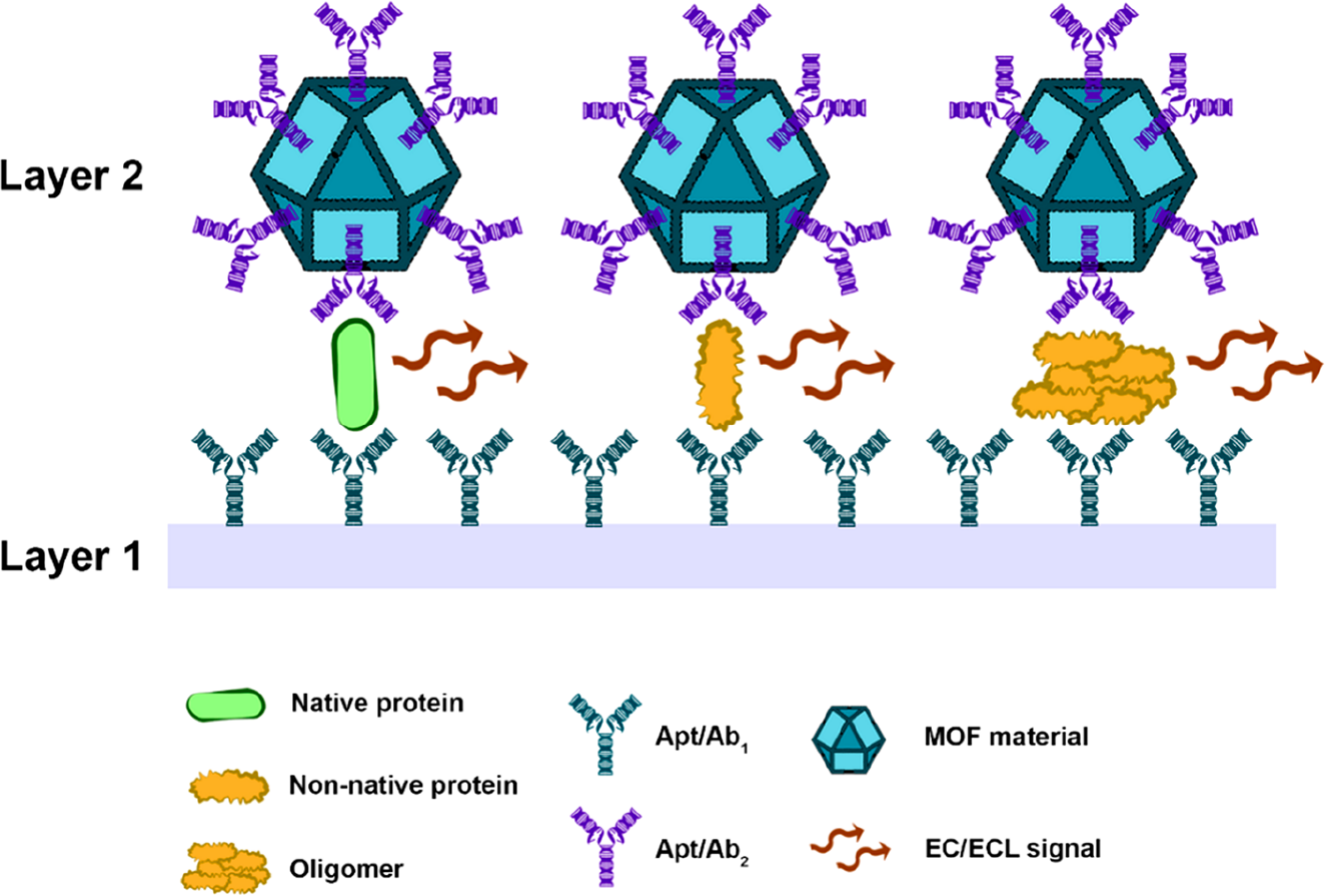
Scheme of the general architecture of a “sandwich-type” MOF-based sensor for amyloid precursor detection. Figure prepared with icons sourced from ref (3).

Most of the MOF-based amyloid sensors discussed in this Review exhibit a sandwich-type design (Figure 4). These sensors are composed of two layers, where either one or both can contain an MOF. Layer 1 can be assembled by coating a glass carbon electrode surface with a modified graphitic carbon nitride nanosheet (*g*-C_3_N_4_) or an MOF functionalized with a first aptamer (Apt_1_) or antibody (Ab_1_) against the target of interest. Then, the target protein/peptide or oligomer thereof binds to layer 1. Subsequently, a second MOF-based layer (layer 2), labeled with a second antibody/aptamer, binds the target analyte, which becomes “sandwiched”. Depending on the components employed, an electrochemical (EC) or electrochemiluminescent (ECL) signal is generated in a concentration-dependent fashion, allowing a precise quantification.^48^ For classical examples of this assembly, we direct the reader to the following past publications: Cu-Al_2_O_3_-*g*-C_3_N_4_-Pd Ab_1_ (layer 1)/UiO-66@PANI-MB Ab_2_ (layer 2) and Ru(bpy)_3_^2+^-Zn-oxalate-MOF-Ab_1_ (layer 1)/Au-NiFe-MOF-Ab_2_ (layer 2) (Ru(bpy)_3_^2+^-Zn-oxalate-MOF-[Ru(bpy)_3_][Zn_2_(C_2_O_4_)_3_] and NiFe-MOF–Ni_3_[Fe(CN)_6_]_2_·10H_2_O).^49^ A full list of “sandwich-type” and other types of MOF-based sensors are described in Tables 1 and 2.

**Table 1:** MOF-Based Sensors for Direct Detection of the Alzheimer’s Disease-Associated Amyloid-β Peptide^a^

sensor	detection method	Aβ form	linear detection range (nM)	detection limit (nM)	pH	sample incubation time	temp. (°C)	stability (readings)^b^	storage	recovery rate (%)	ref
Ab_2_/Au@Fc-Zn-MOF	EC	presumably Aβ_1–42_	2.2 × 10^–5^–22	6.7 × 10^–6^	7.4	2 h	37	10	n/a^c^	>90 (serum)	(52b)
Cu-Al_2_O_3_-*g*-C_3_N_4_-Pd Ab_1_/UiO-66@PANI-MB Ab_2_	EC	Aβ (not specified)	2.2 × 10^–6^–22	7.3 × 10^–7^	6.8	2 h	RT	5	7–30 days, 4 °C	>98 (serum)	(54)
AuNPs@CuMOF/SD	EC	Aβ oligomers	5 × 10^–7^–5 × 10^–3^	2.5 × 10^–7^	7.4	30 min	37	7	29 days, 4 °C	>96 (serum)	(55)
*g*-C_3_N_4_@AuNPs Ab_1_/PdNPs@ MIL-53-NH_2_(Al) Ab_2_	ECL	Aβ_1–42_	2.2 × 10^–6^–11	7.6 × 10^–7^	7.4	1.5 h	RT	14	n/a	>95 (serum)	(57)
*g*-C_3_N_4_-Ru@MOF/S_2_O_8_^2–^	ECL-RET	Aβ (not specified)	2.2 × 10^–6^–110	8.7 × 10^–7^	7.4	>1 h	n/a	9	n/a	>99 (serum)	(58)
[Ru(bpy)_3_]^2+^-Zn-oxalate-MOF-Ab_1_/Au-NiFe-MOF-Ab_2_/TPA	ECL-RET	Aβ (monomer)	2.2 × 10^–5^–11	3.06 × 10^–6^	7.4	>2 h	4	3	n/a	>99 (CSF)	(49a)
[Ru(bpy)_3_]^2+^-NH_2_-UiO-66-Ab_1_/Ab_2_-MIL-101(Cr)@Au-MoS_2_ QDs/TPA	ECL-RET	Aβ (not specified)	2.2 × 10^–6^–11	7.35 × 10^–7^	7.5	>2 h	4	13	7 days, 4°C	>96 (CSF)	(56)
AuNPs/Fe-MIL-88NH_2_	ECL	Aβ oligomers	10^–5^–10^–3^	7.1 × 10^–7^	7.4	1.5 h	37	n/a	16 days	>98 (serum)	(62)
CuO/*g*-C_3_N_4_+MoS_2_ QDs@Cu NWs	PEC	Aβ oligomers	1 × 10^–5^–5 × 10^2^	5.79 × 10^–6^	7.4	1 h	37	10	14 days	>98 (serum)	(65)
ZIF-8/Fc	EC	Aβ oligomers	10^–2^–10^4^	n/a	7.4	15 min	RT	n/a	n/a	>97 (CSF)	(67)
ZnO-Co_3_O_4_	EC	Aβ monomers/oligomers/fibrils	5–150	3.5 (*in vitro*); 1.58 (rat CSF)	5.0	15 min	25	n/a	n/a	>92% (rat CSF)	(73)
Cu-BTC/Tb	F	Aβ_1–40_	1–550 (*in vitro*); 5–490 (plasma)	0.3 (*in vitro*); 1 (plasma)	7.4	20 min	RT	n/a	n/a	>95 (serum)	(69)
Ru-MIL-101(Al)-Apt-AuNPs/RecJF	F	Aβ oligomers	10^–3^–10	3 × 10^–4^	7.4	30 min	RT	5	n/a	>93 (serum)	(64)
anti-DNA antibody@MOF (lanthanum-MIL53(Al))/Apt-MB	F	Aβ oligomers	2.2 × 10^–4^–22	8.7 × 10^–5^	7.4	30 min	25	10	7 weeks, RT	>90 (serum)	(64)
ThT@Er-MOF	F	Aβ (not specified)	0–40	0.142	n/a	n/a	n/a	n/a	n/a	n/a	(70)
(luminol-Tb-GMP-Cu)	F	Aβ (not specified)	5 × 10^–2^–80	2 × 10^–2^	7.4	30 min	37	n/a	n/a	n/a	(60)

a EC, electrochemical; ECL, electrochemiluminescence; RET, resonance energy transfer; F, fluorescence; PEC, photoelectrochemical.

b Number of consecutive readings tested.

c n/a: not available.

**Table 2:** MOF-Based Sensors for Disease-Associated Human Amyloid-Forming Proteins^a^

amyloidosis	protein marker	detection method	sensor	detection	linear detection range (nM)	detection limit (nM)	ref
Parkinson’s disease	α-synuclein	ECL	α-syn/MOF-1	aptamers	2.43 × 10^–6^–4.86 × 10^–4^	4.2 × 10^–7^	(20)
			α-syn/MOF-2	aptamers	1.36 × 10^–6^–2.43 × 10^–4^	3.8 × 10^–7^	(20)
Parkinson’s disease	α-synuclein	α-synuclein	Tb-MOF@Pt-Aptamer	aptamers	10^–1^–10^4^	4 × 10^–2^	(84)
localized insulin-derived amyloidosis	insulin	ECL-RET	Au@Pb-β-CD-Ab_1_/chitosan-Ru(bpy)_3_^2+^-Si NPs-Ab_2_/K_2_S_2_O_8_	antibodies	1.7 × 10^–5^–1.7	7.3 × 10^–7^	(86)
		ECL-RET	UiO-67-Ru(bpy)_3_^2+^-Ab_1_/Au@SiO_2_-Ab_2_/TPA	antibodies	4.3 × 10^–4^–8.6	1.7 × 10^–4^	(87)
		F	Gd-H_3_tpta/aptamer/FAM-P	aptamers	up to 1.72 × 10^3^	1.2	(89)
		MS	Mag MOF@Au@HIA	aptamers	0.9–17	0.17 (*in vitro*); 0.34 (serum)	(90)
		E	calcinated CoNi-ZIF@CoFePBA	aptamers	1.7 × 10^–6^–17	1.6 × 10^–6^	(96)
medullary thyroid cancer	procalcitonin	ECL	MIL-101(Al):Ru-PEI-Au-Ab_1_/Fe_3_O_4_@PDA-Cu_*x*_O-Ab_2_	antibodies	3.5 × 10^–5^–6.9	1.24 × 10^–5^	(92)
		ECL	Au-AgCys-HGC-Ab_1_/Ab_2_-Au-PTCA@ZIF-67/S_2_O_8_^2–^	antibodies	6.9 × 10^–7^–6.9	2.53 × 10^–7^	(91)
pituitary prolactinoma	prolactin	F	Pr-MOF nanofibers	direct interaction with MOF	up to 8	0.01	(93)
hereditary non-neuropathic systemic amyloidosis	lysozyme	E	493-MOF-BA	aptamers	3.4 × 10^–4^–6.8 × 10^–2^	2.4 × 10^–4^	(95)
ApoA4 amyloidosis	apolipoprotein A4	E	HRP-Strept-Biotin-Ab-Apo-A4-ABA/ZIF-8@N-Gr/GCE	antibodies	3.2 × 10^–3^–6.6	1.8 × 10^–3^	(91)

a E, electrochemical; ECL, electrochemiluminescence; RET, resonance energy transfer; F, fluorescence; MS, mass spectroscopy.

## Sensors for Alzheimer’S Disease

### Immunosensors and Aptasensors

Immunosensors are affinity-based devices that use antibodies as the biorecognition element.^50^ The formation of antigen–antibody complexes is highly specific and accurate, translating to the performance of the sensor.^50^ On the other hand, aptasensors use aptamers, single stranded DNA or RNA designed to specifically bind a target of interest, like proteins or peptides, as recognition elements.^51^ Despite a lower affinity toward the target, aptamers usually cost ten times less than antibodies, making them extremely attractive for large-scale sensor production. These sensors are known to be highly sensitive and can detect nanomolar to femtomolar concentrations of biomolecules.

**Figure 5:**
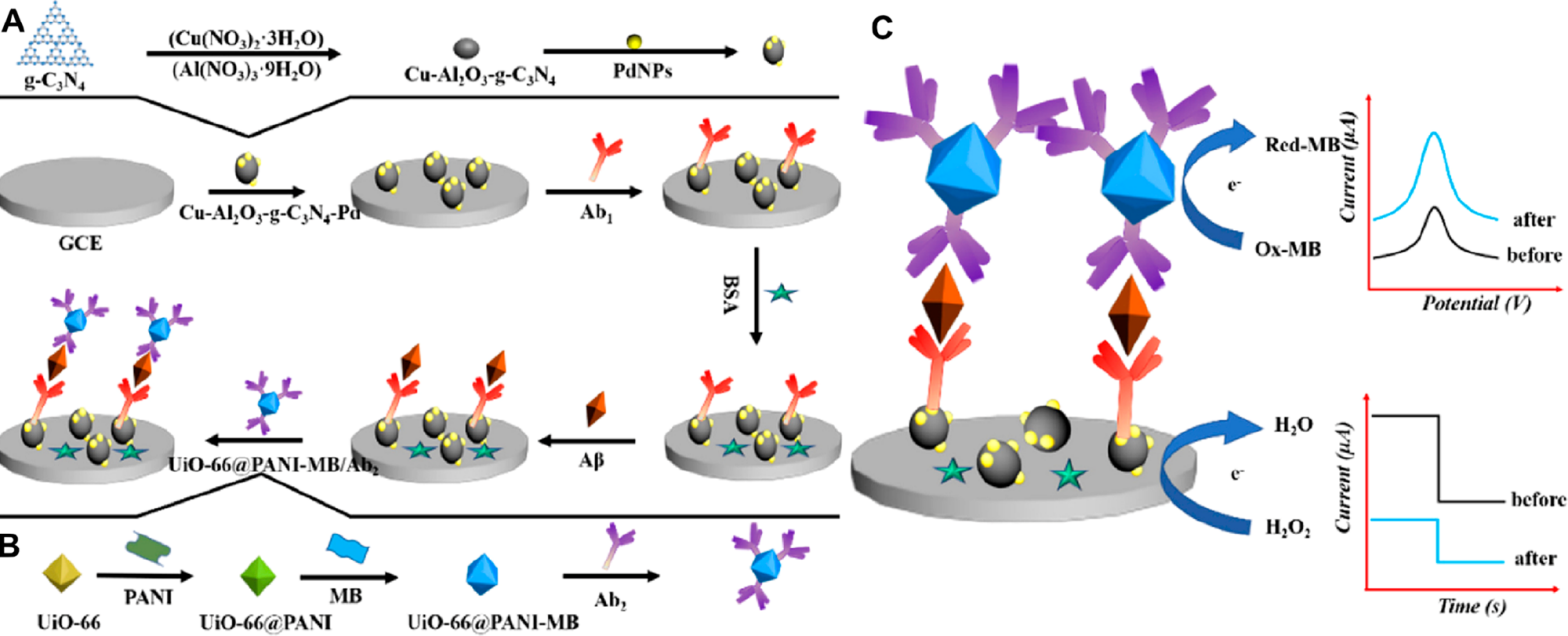
Schematic representation of the biosensor construction: (A) layer 1; (B) layer 2; and (C) detection signals. Adapted with permission from ref (54). Copyright 2019 Elsevier.

Four immunosensor devices based on an electrochemical (EC) signal were created.. Han and co-workers developed a “sandwich” immunosensor for Aβ. The electrochemical signal tag (layer 2 depicted in Figure 4) is ferrocene (Fc) (redox mediator) covalently linked to pendant amine groups within the inner channels of IRMOF-1 (known as MOF-5, [Zn_4_O(bdc)_3_]).^52^ The MOF allowed protection of the Fc, avoiding its leakage in a wide range of pH conditions (3–10), with increased sensitivity. Fc-Zn-MOF was decorated with gold nanoparticles (AuNPs), and an anti-Aβ (Ab_2_) was attached (Ab_2_/Au@Fc-Zn-MOF). The immunosensing interface fabrication (layer 1 depicted in Figure 4) was possible by dispersing amino-terminated polyamidoamine (PAMAM) dendrimers onto a graphene surface, followed by fixation of AuNPs. Finally, anti-Aβ (Ab_1_) was immobilized on the surface. The sensor could detect *A*β quantitatively from 2.2 × 10^–5^ to 22 nM (linear range), with a limit of detection (LOD) of 6.7 × 10^–6^ nM.^52b^ This sensor exhibited high specificity for Aβ (against, for example, the serum-abundant albumin) and Aβ recovery rates of above 90% from simulated human serum (Aβ diluted to real human serum).^52b^ A similar sensor, using aptamers as the recognition element, was proposed by Zhou et al.^53^ Briefly, HKUST-1 was prepared through the reaction of 1,3,5-benzenetricarboxylic acid with CuSO_4_·3H_2_O, yielding a material with high electrochemical signal (Cu^2+^ possesses redox activity) but inherent low conductivity (no additional information related to the MOF identity is provided). To improve signal transduction, HKUST-1 was loaded with AuNPs that allowed the immobilization of an aptamer specific to Aβ oligomers to obtain the electrochemical tag (layer 2: aptamer-tagged AuNPs/HKUST-1 conjugates). Layer 1 consisted of a glass carbon electrode (GCE) decorated with gold nanoflowers (AuNFs) functionalized with the same aptamer. The sensor has a detection limit of 0.45 nM for Aβ oligomers and showed comparable results to the currently used commercial ELISA kits with recovery rates over 97%.^53^ Miao and co-workers presented another sandwich electrochemical dual-signal biosensor prepared using UiO-66 ([Zr_6_O_4_(bdc)_2_]) NPs (100–200 nm) (layer 2) combined with the conductive polymer polyaniline (PANI). PANI has abundant amino groups that were used to immobilize an anti-Aβ antibody (Ab_2_).^54^ The conjugate was loaded with methylene blue (MB, a redox mediator used in electrochemical immunosensing), and the Al_2_O_3_ lattice was doped with Cu (Cu-Al_2_O_3_) and incorporated into graphite carbon nitride sheets (*g*-C_3_N_4_) to yield Cu-Al_2_O_3_-*g*-C_3_N_4_ (layer 1). Subsequently, palladium NPs were introduced to the layer for the immobilization of anti-Aβ (Ab_1_) via Pd-NH_2_. The sensor response to Aβ binding arises from two signals, allowing a very sensitive detection of Aβ. Current amperometry was performed via Cu-Al_2_O_3_-*g*-C_3_N_4_-Pd catalytic reduction of hydrogen peroxide and square wave voltammetry from methylene blue reduction (Figure 5).

Upon optimization (namely, pH and component concentrations), this sensor could detect Aβ in a linear range of 2.2 × 10^–6^ to 22 nM and an LOD of 7.3 × 10^–7^ nM.^54^ In addition, the sensor was able to reach recovery rates of over 98% in simulated human serum.

The last example of electrochemical detection is an “on–off” triple helix switch (THS) sensor coupled to MOFs with a AuNP-labeled signal-displaced probe, named AuNPs@CuMOF/SD (with CuMOF being a copper-based IRMOF-3).^55^ This sensor is highly specific toward Aβ oligomers, with detection by “switch off” when oligomers bind the aptamer and disassemble the THS structure. Aβ oligomers were detected in the range 5 × 10^–7^ to 5 × 10^–3^ nM, with an LOD of 2.5 × 10^–7^ nM, which make this the most sensitive Aβ oligomer sensor discussed in this Review. Along with good recovery rates in artificial CSF and storage/reproducibility features, it compared well to currently used ELISA methods; however, transformation into batch and portable testing is an obstacle for widespread use.

**Figure 6:**
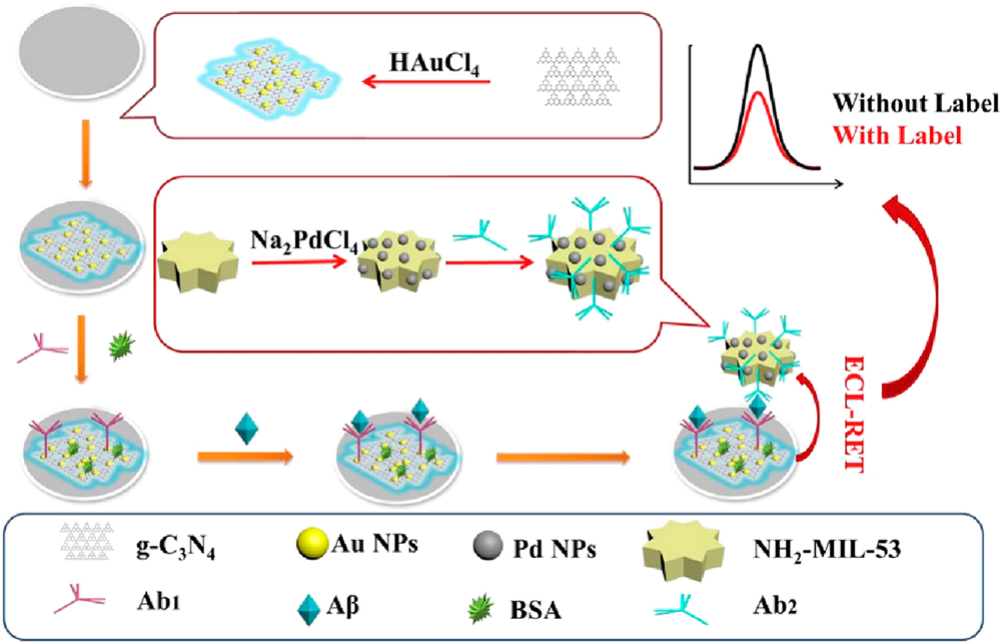
Schematic illustration of an ECL immunosensor. Cao and colleagues used *g*-C_3_N_4_@AuNPs as the donor and PdNPs@NH_2_-MIL-53 as the receptor. Adapted with permission from ref (57). Copyright 2019 Elsevier.

A series of sandwich-type biosensors have been fabricated based on a quenching electrochemiluminescence (ECL) strategy. Based on the detection mechanism, the ECL emission spectra of one layer must significantly overlap with the other’s absorption spectra. ECL-resonance energy transfer (RET) occurs only when the two layers are brought together (<10 nm) by Aβ cross-bridge. Wang and co-workers used [Ru(bpy)_3_]^2+^ cations encapsulated in zinc oxalate MOFs as donor.^49a^ The MOF shielded the chromophores from solvent molecules and led to a high Ru emission efficiency. [Ru(bpy)_3_]^2+^-Zn-oxalate-MOF, conjugated with a first Aβ antibody (Ab_1_), was coated to a glassy carbon electrode surface (layer 1). The authors observed that both AuNPs and NiFe-based nanocube MOFs contributed to the reduction (absorption) of the ECL signal, and thus Au@NiFe MOFs were used as an acceptor (layer 2). This sensor was specific to Aβ monomers, failing to produce a signal in the presence of Aβ oligomers or fibrils with a detection range from 2.2 × 10^–5^ to 11 nM (LOD: 3.06 × 10^–6^ nM), and recovery rates over 99% in simulated CSF. Another ECL sensor was fabricated with [Ru(bpy)_3_]^2+^ cations encapsulated in NH_2_-UiO-66 labeled with primary antibodies (Ab_1_), acting as luminophore, and MoS_2_ quantum dots combined with MIL-101 labeled with secondary antibodies (Ab_2_) to quench the ECL signal. This sensor specifically detected Aβ from 2.2 × 10^–6^ to 11 nM, with a detection limit of 7.35 × 10^–7^ nM, presenting similar results to those of the commercially available ELISA Aβ detection kit, with recovery rates over 96%, in both artificial and real human CSF.^56^ A similar performance for detection of Aβ was reached by a sandwich immunosensor using *g*-C_3_N_4_@AuNPs as the donor and PdNPs@MIL-53-NH2 ([Fe(OH)(bdc-NH_2_)]·H_2_O) as the receptor (Figure 6).^57^

**Figure 7:**
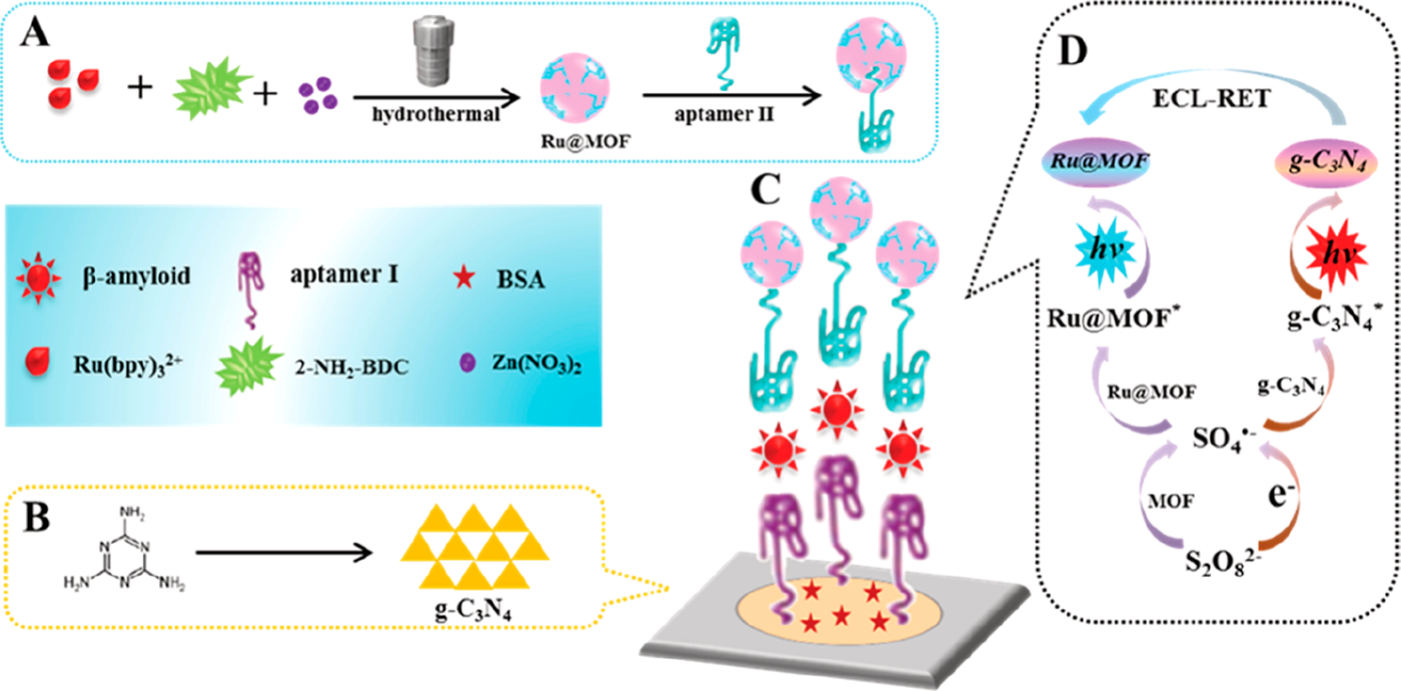
Synthesis of aptamer II–Ru@MOF-based ECL-RET acceptor. (A) Synthesis of aptamer II–Ru@MOF signal probe. (B) Fabrication of *g*-C_3_N_4_ NS. (C) Dual-wavelength ratiometric ECL sensor. (D) Proposed detection mechanism. Adapted with permission from ref (58). Copyright 2019 American Chemical Society.

Jia’s research group designed a more elaborated ratiometric (measures changes in the ratio of two signals, ECL_460 nm_/ECL_620 nm_) ECL-RET aptasensor between a *g*-C_3_N_4_ nanosheet and Ru@MOF.^58^ The material was prepared by loading [Ru(bpy)_3_]^2+^ into IRMOF-3 ([Zn_4_O(bdc-NH_2_)_3_], where H_2_bdc-NH_2_ stands for 2-amino-aminoterephthalic acid)^59^ to form a highly luminescence-functionalized MOF. This layer in combination with the aptamer *g*-C_3_N_4_ NS one was responsive toward Aβ intercalation from 2.2 × 10^–6^ to 110 nM (LOD of 8.7 × 10^–7^ nM), denoting the widest detection range of MOF-based biosensors to date (Figure 7). Moreover, H_2_bdc-NH_2_ organic linkers catalyzed the conversion of S_2_O_8_^2–^ (coreactant added to the buffer medium) to SO_4_^•–^ which enhanced the ECL signal at 620 nm of [Ru(bpy)_3_]^2+^. This sensor is selective toward Aβ, with recovery rates over 99% in simulated human serum. The ECL-RET efficiency from *g*-C_3_N_4_ NS to Ru@MOF was ascribed to the *g*-C_3_N_4_ NS ECL signal intensity overlap with the UV–vis absorption band of Ru@MOF.

Recently, Liu and co-workers designed a ratiometric fluorescence probe based on a luminescent coordination polymer.^60^ This probe was composed of Tb^3+^ cations as the metal center, Cu^2+^ ions as cofactors for the fluorescence silence, with guanine monophosphate as the bridging linker, and luminol as an auxiliary linker. The high binding affinity of Aβ toward Cu^2+^ causes an emission enhancement on the probe, while the emission of luminol remains constant, thus acting as a reference. The Aβ determination was performed by a fluorescence ratio between luminol and Tb^3+^, with a sensitivity of 20 pM.

In 2019, Wang and co-workers used the ECL signal emission probe (layer 2) to detect Aβ_1–42_.^61^ An analogue of luminol, *N*-(aminobutyl)-*N*-(ethylisoluminol) (ABEI), was cross-linked with H_2_bdc-NH_2_ and employed as an organic linker to prepare Co-MOFs/ABEI with a strong ECL signal. In addition, Co-MOFs accelerated the conversion of the coreactant H_2_O_2_ to reactive oxygen species (ROS) that enhanced the ABEI ECL signal. Aβ_1–42_ was detected from 2.2 × 10^–6^ to 22 nM, with a detection limit of 6.7 × 10^–7^ nM. The recovery rates in simulated serum were over 96%, and Aβ_1–42_ was detected in real human serum samples collected from hospital patients.

The last ECL sensor consists of a AuNP-enriched Fe-MIL-88NH_2_ MOF attached via Au–N bonds to an indium tin oxide (ITO)-coated glass surface. The Aβ-specific aptamer is attached to the AuNPs via Au–S bonds. Using luminol as a reporter, the signal was analyzed by cyclic voltammetry and electrochemical impedance spectroscopy.^62^ The usage of the MOF drastically increased the ECL signal over the bare ITO surface. This sensor allowed the specific detection of Aβ oligomers (monomers and fibers generated only a slight signal, while other species, such as α-synuclein oligomers, were not detected), with a linear range of 10^–5^ to 10^–3^ nM and LOD of 7.1 × 10^–7^ nM. Furthermore, it achieved a recovery rate in real human serum above 98%, with a clinically competitive measurement time of 1.5 h.

For detection of Aβ oligomers, a photoluminescent sensor based on MIL-101(Al) doped with [Ru(bpy)_3_]^2+^ was reported.^63^ The MOF was postsynthetically functionalized with an Aβ oligomer aptamer/AuNPs for target recognition. Without Aβ, the ruthenium signal is quenched by the aptamer-AuNPs. When bound to Aβ oligomers, the system is “turned on”, with the fluorescent signal amplified by the action of enzyme RecJF exonuclease that acts on the aptamer/Aβ complex, excising it from the Ru@ MIL-101(Al) and eliminating the quenching effect. In addition, this enzyme allows for the recycling of the analyte. In terms of performance, the optimum reaction time of this sensor is 30 min, making it very convenient for clinical use. In addition, it is specific for Aβ oligomers (i.e., over other blood components, such as cholesterol or albumin) in simulated serum, with a linear detection range from 10^–3^ to 10 nM, an LOD of 3 × 10^–4^ nM, and recovery rates over 93%.

Making use of label-free aptasensor based on a lanthanum-modified MIL-53(Al), for cost reduction production, Ren et al.^64^ prepared an Aβ oligomer sensor coined of anti-DNA antibody@MOF (lanthanum-MIL53(Al))/Apt-MB. Interestingly, a simple heat treatment rescued the Aβ-bound aptamers, making them available for further measurements, further improving the simplicity and cost effectiveness of the sensor.^64^ The high sensitivity (ranging from 2.2 × 10^–4^ to 22 nM, with an LOD of 8.7 × 10^–5^ nM), specificity, reusability, and stability showed that this sensor is an excellent platform for Aβ oligomers. However, large-scale production may hinder its use beyond the laboratory setting: this sensor, CuO/*g*-C_3_N_4_ + MoS_2_ QDs@Cu NWs, is a two-component sensor anchored on an ITO surface.

The CuO/*g*-C_3_N_4_ constitutes the photoactive material, while MoS_2_ QDs@Cu NWs (QDs = quantum dots; NWs = nanowires) act as a signal amplifier with intrinsic peroxidase-like activity. The Cu NWs result from the *in situ* pyrolysis of HKUST-1 with dicyandiamide.^65^ The binding of Aβ oligomers to aptamers dissociates the latter from dsDNA, allowing the recovery of photocurrent. This is thus an on–off–on sensor. Detection is highly specific for Aβ oligomers over monomers, fibrils, or other proteins, such as lysozyme or insulin. With a very broad detection range (1 × 10^–5^–5 × 10^2^ nM) and low LOD (5.79 × 10^–6^ nM), as well as good performance with human serum, this sensor constitutes a good example of how the MOF properties can be modulated to improve their sensing capabilities.

### Other Sensors

**Figure 8:**
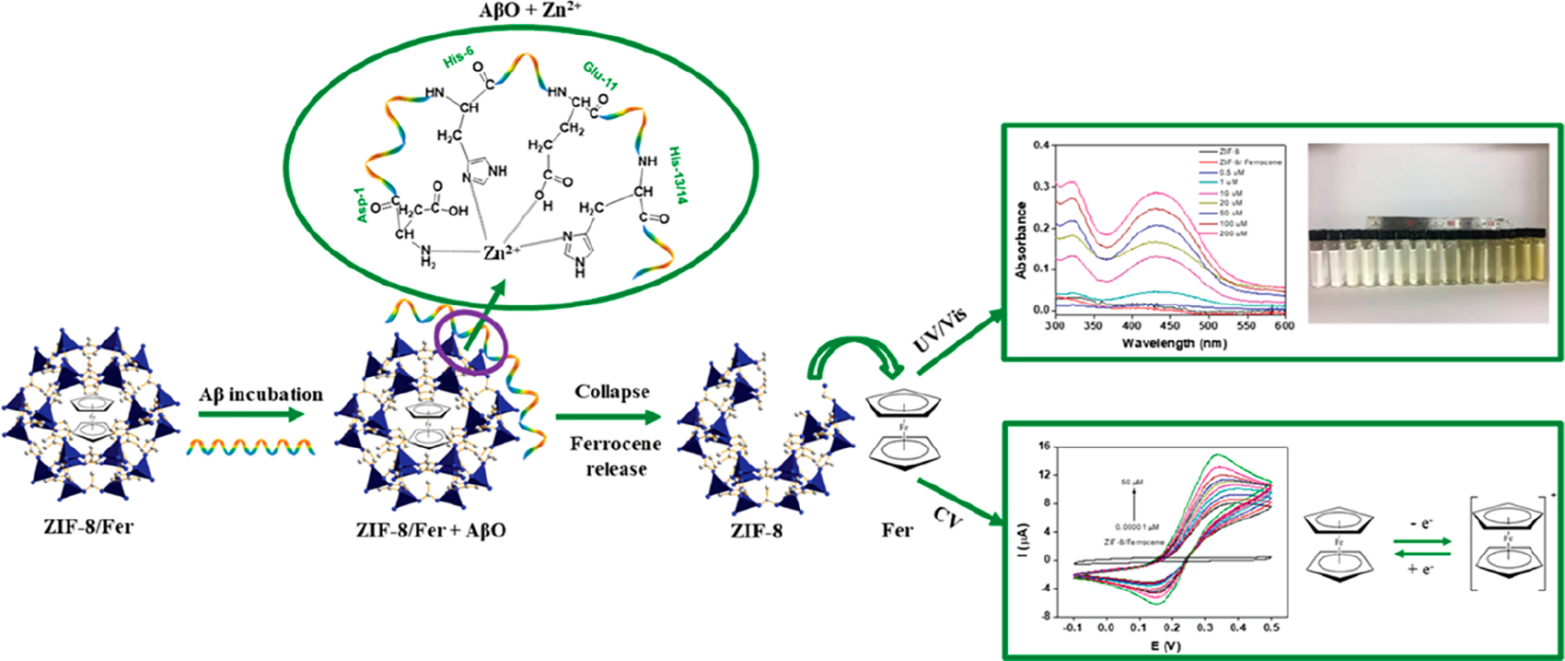
ZIF-8/Fc was used for optical or electrochemical detection of Aβ oligomers. Aβ affinity toward zinc clusters causes the collapse of the ZIF-8 framework, subsequently releasing ferrocene in a concentration-dependent manner. Adapted with permission from ref (67). Copyright 2019 American Chemical Society.

Aβ has strong affinity for divalent metal cations, likely through its *N*-terminal side present in the imidazole side chains of histidine H6, H13, and H14.^66^ Taking advantage of these interactions, a few Aβ MOF sensors housing these transition metals as coordination centers were developed. Qin and co-workers developed a ZIF-8-based sensor for Aβ oligomers by encapsulating ferrocene (Fc) within the pores (ZIF-8/Fc).^67^ The binding of Aβ oligomers to the framework zinc centers leads to the disassembly of ZIF-8 and consequent release of ferrocene. This release is dependent on oligomer concentration and may be optically or electrochemically detected (Figure 8). Optically, a linear range up to 10^4^ nM Aβ oligomers could be detected by UV/vis absorption of the released ferrocene, with the novelty of possible adaptation for smartphone reading from 200 to 1000 μM. This concentration range falls within a pathological range, being significantly higher than the expected physiological one.^68^ Still, Aβ oligomers are detected much more precisely by electrochemical methods with linear ranges around 0.01–10^4^ nM. This sensor exhibits a good shelf life (up to a month) and specificity toward oligomers (over monomers or fibrils; the former likely does not displace zinc from the ZIF) and performed well in artificial CSF (recovery rate over 97%). Technical challenges may, however, arise with physiological samples because of the presence of proteins with zinc binding motifs that may mimic Aβ oligomers in the degradation of ZIF-8/Fc.

Liu and co-workers used Cu-BTC/Tb (Cu-BTC also known as HKUST-1), a lanthanide-functionalized fluorescent MOF sensor for the detection of Aβ_1–40_.^69^ Cu-BTC/Tb was prepared by postsynthetic inclusion of Tb^3+^ in the prepared Cu-BTC. In contrast to the strong fluorescence of Tb-BTC (a different crystalline material using the same organic linker but with Tb^3+^), the emission peaks of Cu-BTC/Tb at 488, 545, 583, and 621 nm in the visible region associated with the ^5^D_4_ → ^7^F_*J*_ (*J* = 6, 5, 4, and 3) transitions of Tb^3+^ are very weak. The fluorescence variation of Cu-BTC/Tb with different molar proportions of Cu^2+^ to Tb^3+^ was attributed to the paramagnetic nature of Cu^2+^ which generated the luminescence quenching of Tb^3+^ by the relaxation of the excitation energy by a nonradiative pathway. Aβ presumably sequesters copper ions from the framework, eliminating the quenching effect over Tb^3+^ and consequently producing fluorescence. The sensor is stable up to 10 days with no observable fluorescence decrease and was optimized for a maximum signal with a Cu^2+^:Tb^3+^ molar ratio of 1:5. *In vitro* studies show that Cu-BTC/Tb could specifically detect Aβ_1–40_ in a linear range from 1 to 550 nM with an LOD of 0.3 nM. Notably, the sensor is highly specific toward Aβ_1–40_. Aβ_1–42_, whose typical concentrations are one tenth of Aβ_1–40_, was only detected for the highest tested concentrations, while aggregated Aβ yielded no signal. These differences likely stem from different propensities of Aβ species to sequester Cu^2+^. Other plasma components, such as albumin and fibrinogen, did not interfere with the Aβ_1–40_ detection. Furthermore, using simulated real plasma samples, this sensor exhibited a linear detection range for 5–490 nM Aβ_1–40_ (LOD 1 nM) and recovery rates over 95%.^69^

ThT@Er-MOF is a luminescent MOF-based sensor for three Alzheimer’s markers (Aβ, acetylcholine, and PSEN1 mutation-prone DNA region)^70^ based on an erbium-based MOF ([Er(L)(DMF)_1.27_] where H_3_L stands for terphenyl-3,4″,5-tricarboxylic acid, postsynthetically modified with the benzothiazole thioflavin-T (ThT). Analyte quantification was based on ratiometric fluorescent detection of the lanthanide and ThT (amyloid fluorescent dye, which forms G-quadruplex complexes when binding to DNA, increasing its fluorescence emission).^71^ Thus, ThT@Er-MOF allows the direct (Aβ and acetylcholine) or indirect (PSEN1 mutation prone DNA sequences) detection of AD. In particular, ThT@Er-MOF detected Aβ up to 40 nM in simulated CSF, with an LOD of 0.142 nM, while exhibiting high selectivity against other hypothalamus circulating proteins such as corticotropin releasing hormone.^70^ Despite better sensitivities of other sensors toward for Aβ (Table 1), the ThT@Er-MOF detection of three separate Alzheimer biomarkers strengthens the versatility of this porous material for sensing purposes. CSF levels of Aβ_1–42_ correlate with triglyceride levels. Taking advantage of the MOF loading abilities, a sensor for triglycerides in blood level was developed based on fiber optics consisting of birefringent interferometer *in situ* immobilization of a ZIF-8/lipase complex.^48,72^ Depending on the waist diameter of the sensor, different LODs were achieved. For example, for a 7 μm tapered region, triglycerides could be detected from 0 to 50 nM (LOD of 0.23 nM) with high specificity and, more importantly, using blood samples.^48^

### Inhibitors of Aβ Aggregation

There are a handful of cases in which MOFs are employed to inhibit aggregation. Many studies (as reviewed by Jokar et al.)^74^ show effective inhibition of aggregation, either by metal chelation and subsequent inhibition of metal-dependent aggregation (as discussed before, Aβ shows affinity to divalent metal cations)^66c,75^ or by inhibiting the formation of amyloid β-sheets (the so-called β-sheet breakers). Typically, β-sheet breakers belong to several categories such as organic molecules, peptides, antibodies, and NPs (e.g., carbon nanotubes or polymeric). Some, like polyphenolic compounds from grapeseed extracts or the antibody solanezumab, have entered into different phases of human clinical trials.^76^ In this context, MOFs are emerging compounds that may be effective in inhibiting Aβ aggregation.

Oxidation of Aβ is a potential AD therapy because, generally, oxidation of amyloid precursors leads to impaired aggregation and even mature fibril disassembly.^77^ The most employed photo-oxidizing agents have, however, a number of intrinsic drawbacks. For example, free porphyrins tend to aggregate and become inactive because of auto-photo-oxidation phenomena and still lack the ability to target specific moieties.^78^ The use of porphyrinic ligands in MOFs can tackle these issues. The Porous Coordination Network 224 (PCN-224, [Zr_15_(TCPP)_3_(μ3-OH)_16_(OH)_20_(H_2_O)_4_]) can generate singlet oxygen by near-infrared (NIR) photoinduction that acts on Aβ_1–42_ greatly reducing its aggregation and, subsequently, the cytotoxicity of its aggregates as assessed on PC12 cells.^79^ This inhibitory effect is dependent on photoinduction (i.e., no aggregation inhibition was recorded by the presence of unirradiated PCN-224), directly correlating with illumination intensity as well as the concentration of the MOF *in vitro* (up to 100 μg mL^–1^). Compared with other aggregation inhibitors, such as organic compounds or photosensitizers, the use of this MOF-based strategy has important advantages. This Zr^4+^-MOF has good water stability, biocompatibility, high degree of singlet oxygen generation (conferred by combination of the high porphyrinic content and overall porosity), and the ability to surpass the blood–brain barrier because of the nanometric particle size. MOF NPs were hydrothermally synthesized to an average size of 70 nm. Overall, and despite the lack of crucial *in vivo* studies, this strategy is promising as a potential MOF-based noninvasive phototreatment for AD, with the use of NIR radiation allowing a greater penetration efficiency within the brain tissue (as compared to other phototreatments using visible radiation).^79^

**Figure 9:**
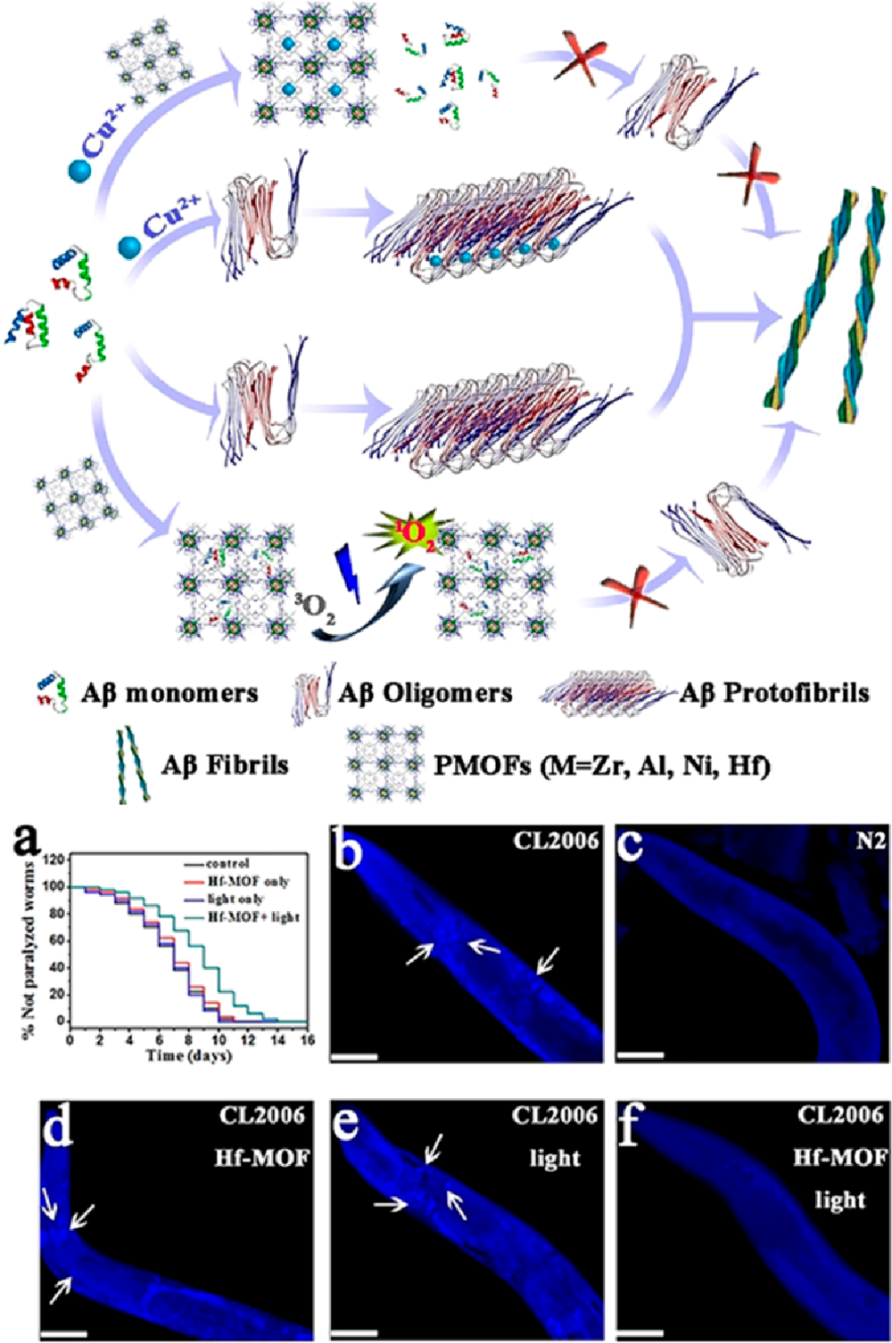
Schematic representation of a possible mechanism for Aβ aggregation inhibition by porphyrin-based MOFs. Oxidation of Aβ monomers through singlet oxygen combined with copper harnessing greatly reduces peptide aggregation. Effect of LPFFD-PCN-224(Hf) on life span (a) and amyloid plaque formation (white arrows, b–f) in a transgenic A*β C. elegans in vivo* model: (b) CL2006 transgenic worm; (c) N2 wild type worm; (d) CL2006 with LPFFD-PCN-224(Hf); (e) CL2006 with light; and (f) CL2006 with LPFFD-PCN-224(Hf) and light. Adapted with permission from ref (82). Copyright 2019 John Wiley & Sons.

Yu and co-workers deepened the use of porphyrinic MOFs against Aβ aggregation (Figure 9, top) by preparing other materials with the same ligand of PCN-224(Zr), but by modifying the metal element. PCN-224(Hf) along with two other porphyrin-based MOFs, Al-CP {[(AlOH)_2_H_2_TCPP])^80^ and Ni-CP ([Ni3(Ni-HTCPP)_2_(μ_2_-H_2_O)_2_(H_2_O)_4_(DMF)_2_]·2DMF},^81^ were tested on Aβ_1–40_.^82^ The aromatic rings of the ligands, the porosity, and the availability of coordinating sites at the metallic centers (that can coordinate to the Aβ histidine residues) allow the MOF to be easily enriched in Aβ. In this context, the Ni-CP sequestration of Aβ_1–40_ was slightly higher than that of the others. PCN-224(Hf) exhibited, however, the highest degree of singlet oxygen generation by photoinduction and was selected for subsequent assays.^82^ In addition to the photo-oxidation effect, it was expected that the porphyrin-based linkers could chelate divalent cations that potentiate Aβ aggregation.^54,69,75^ PCN-224(Hf) was functionalized with the specific β-sheet breaker peptide iAβ5 (H-Leu-Pro-Phe-Phe-Asp-OH trifluoroacetate salt = LPFFD), labeled as LPFFD-PCN-224(Hf).^82^ LPFFD significantly increased Aβ enrichment: using an A*β C. elegans in vivo* model, with LPFFD-PCN-224(Hf) being able to reduce Aβ amyloid content (as assessed by thioflavin-S staining) and, more importantly, rescue paralysis/motility impairment induced by Aβ aggregation (Figure 9a–f).^82^ This LPFFD-PCN-224(Hf) constitutes, thus, a prime example antiamyloid therapy in animal models.

Another porphyrin-based MOF was employed by Wang et al. to study the photothermal and photo-oxygenation inhibition of the Aβ_42_ aggregation.^83^ In their work, PCN-222 nanosheets (prepared by the assembly of Zr_6_O_8_ clusters with 5-, 10-, 15-, and 20-tetrakis(4-carboxyphenyl)porphyrin) were immobilized with indocyanine resulting in the PCN-222@ICG composite nanoprobe. This nanoplatform showed a quick response to temperature change (from 25 to 45 °C), producing singlet oxygen in the near-infrared (NIR) region. The Aβ_42_ aggregation was measured by dynamic light scattering and TEM for a period of 24 h. The photoactivated nanoprobe showed a strong inhibition effect on Aβ_42_ aggregation, with aggregates of only 90 nm after 24 h when compared with the ∼1000 nm aggregates observed for the untreated sample. This nanoprobe further exhibited a high permeability for the BBB barrier evaluated on a brain-on-a-chip module.

## Sensors for Parkinson’s Disease

The presence of α-synuclein (α-syn) has been highlighted as one of the agents causing Parkinson’s disease. It is, therefore, important to develop diagnostics and treatments to target this specific oligomer. Recently, Miao and co-workers have prepared a luminescent MOF exhibiting a “turn-on” effect for the noninvasive monitoring of α-syn.^84^ An aptamer supported on Pt nanoparticles was bound to the Tb-MOF (prepared using 3,3‴-dihydroxy-2′,2″,5′,5′′-tetramethyl-[1,1′:4′,1′′:4′′,1‴-quaterphenyl]-4,4‴-dicarboxylic acid as the organic linker). In the presence of α-syn in the gut, the aptamer can recognize the oligomer and selectively bind it. The Pt-aptamer/α-syn complex is released to the gut, leading to “turn-on” in fluorescence of the Tb-MOF probe. Because the probe maintains its stability alongside the GI track and can be drained in the feces, this results in a probe capable of a noninvasive detection of the α-syn oligomer.

**Figure 10:**
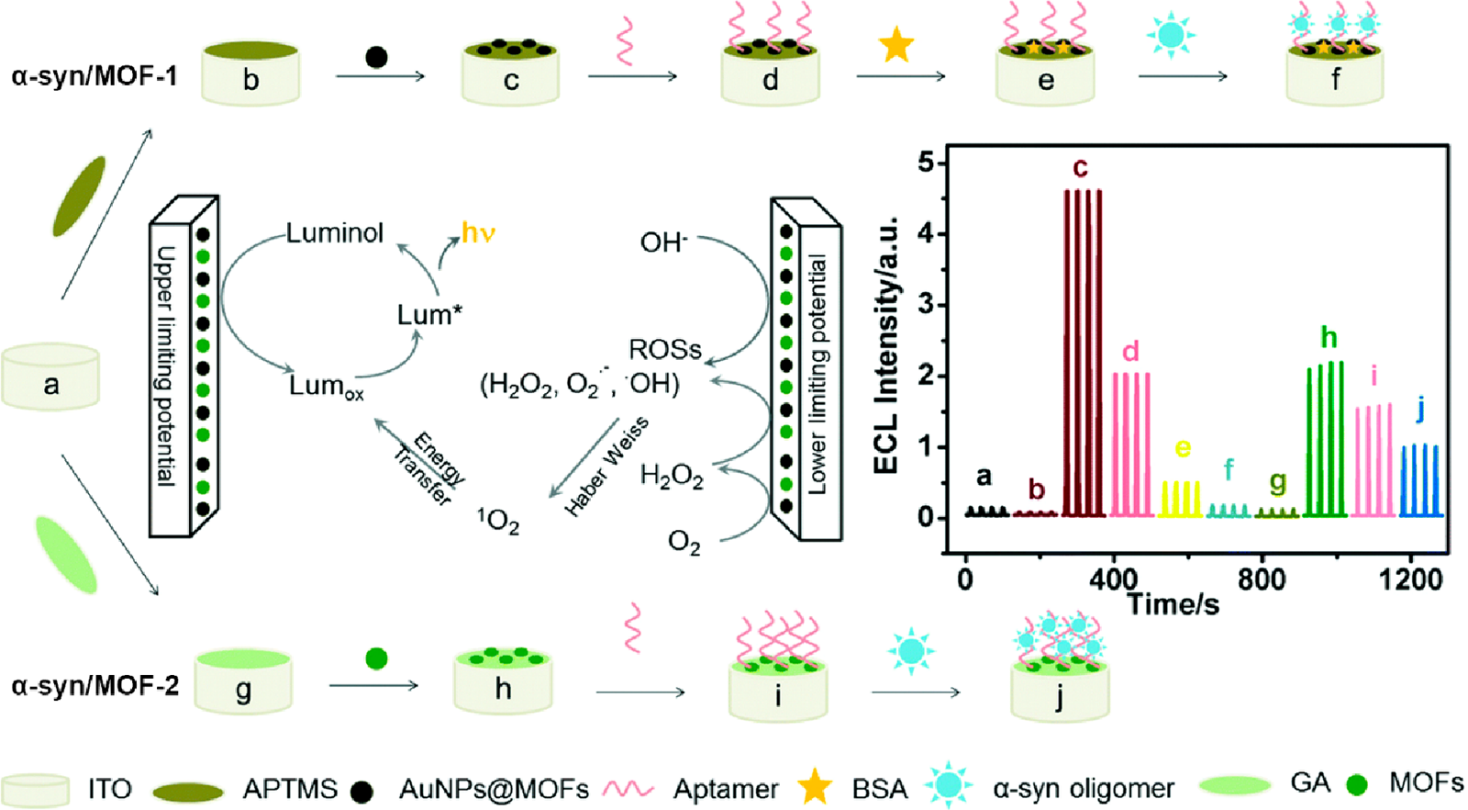
Diagram of the fabrication of α-syn/MOF-1 and α-syn/MOF-2 sensors as well as their sensing mechanism. The inset graph indicates the ECL behavior of each component at different stages: (a) ITO; (b) ITO with hydrolyzed 3-aminopropyl-trimethoxysilane (APTMS); (c) ITO/APTMS/AuNPs@MOFs; (d) ITO/APTMS/AuNPs@MOFs/aptamers; (e) α-syn/MOF-1; (f) α-syn/MOF-1 after *α-*synuclein oligomer binding; (g) ITO with glutaraldehyde (GA); (h) ITO/GA/Cu-MOFs; (i) α-syn/MOF-2; and (j) α-syn/MOF-2 after α-synuclein oligomer binding. Adapted with permission from ref (20). Copyright 2020 Royal Society of Chemistry.

In addition to the nonspecific α-synuclein oligomer detection by an Aβ MOF sensor (AuNFs-AuNPs/Cu-MOFs, as previously discussed),^53^ two variations of specific ECL aptamer-MOF sensors have been proposed to date.^20^ Both sensors rely on what is mentioned as a Cu-MOF that was, unfortunately, barely characterized by the authors. Luminol was employed as the luminescent reagent, with an ITO coating the surface (Figure 10).

One of the sensors (for simplicity herein denoted α-syn/MOF-1) employs Cu-MOFs doped with AuNPs that are covalently bonded to the organic linker. The AuNPs@Cu-MOFs were then physically immobilized on the ITO surface. An aptamer was finally added through Au–S bonds.^20^ As an alternative, a strategy relying solely on the Cu-MOF to promote the ECL signal (i.e., without AuNPs) was developed (herein termed α-syn/MOF-2). The MOFs were grafted onto the ITO surface by glutaraldehyde cross-linking, while the aptamer was directly linked to the MOF by 1-ethyl-3-(3-(dimethylamino)propyl)carbodiimide/*N*-hydroxysuccinimide (EDC/NHS).^20^ As expected, and despite the fact that α-syn/MOF-1 exhibited a stronger ECL signal (because of the AuNPs), both could be applied for detection of α-synuclein oligomers. For both sensors the optimum detection temperature was 35 °C, with a measuring time from 1 to 1.5 h, which is well suitable for clinical laboratory setting. For α-syn/MOF-1, the α-synuclein oligomer detection range goes from 2.43 × 10^–6^ to 4.86 × 10^–2^ nM (LOD of 4.2 × 10^–5^ nM), while for α-syn/MOF-2 it is 1.35 × 10^–6^ to 2.43 × 10^–2^ nM (LOD of 3.8 × 10^–5^ nM). Both sensors exhibit significantly higher sensitivity than other methods, such as ELISA, and specificity toward oligomers (even α-synuclein monomers in concentrations 100 times higher than those employed for oligomers did not elicit an ECL signal).^20^ These sensors may open the possibility for detection of α-synuclein oligomers for PD diagnosis using body fluids such as blood, serum (tested recovery rates over 87%), or even intercellular fluid, eliminating the need for invasive collection of CSF, as currently performed in clinical settings.

## Sensors for Other Amyloidosis

### Localized Amyloidosis Caused by Polypeptide Hormones

Several MOF-based sensors were developed for direct or indirect quantification of polypeptide hormones associated with localized amyloidosis.^85^ Based on the ECL-RET immunodetection principle, two sandwich-type MOF-based insulin sensors have been proposed. Ma and co-workers developed a sensor incorporating an MOF prepared with cyclodextrins (CDs) as organic linkers and lead as the metal center.^86^ CDs are cyclic oligosaccharides with multiple possible metal coordination sites and good aqueous solubility and biocompatibility. The Pb-β-CD MOF, doped with AuNPs for better signal transduction, constitutes the ECL donor. The second layer is composed of chitosan-Ru(bpy)_3_^2+^-SiNPs (chitosan was employed to facilitate functionalization with Ab_2_). Using K_2_S_2_O_8_ as coreactant, an ECL-RET signal is generated, enabling detection of insulin in a linear range from 1.7 × 10^–5^ to 1.7 nM (LOD 7.3 × 10^–7^ nM). Wei and co-workers designed an ECL-RET sensor for insulin based on the encapsulation of [Ru(bpy)_3_]^2+^ into UiO-67, the same strategy used for detection of A*β.*^49a,54,56,87^ UiO-67 increases the ECL signal and has larger pores than UiO-66,^88^ which promotes a much more efficient encapsulation of [Ru(bpy)_3_]^2+^. While UiO-67-[Ru(bpy)_3_]^2+^-Ab_1_ was the ECL-RET donor, Au@SiO_2_-Ab_2_ was used as the ECL-RET acceptor. *In vitro* detection of insulin was possible in the 4.3 × 10^–4^ to 8.6 nM concentration range (LOD of 1.7 × 10^–4^ nM). The sensor was specific for insulin in real human serum samples, enabling detection down to 4.50 nM and a recovery rate of over 98%.^87^

**Figure 11:**
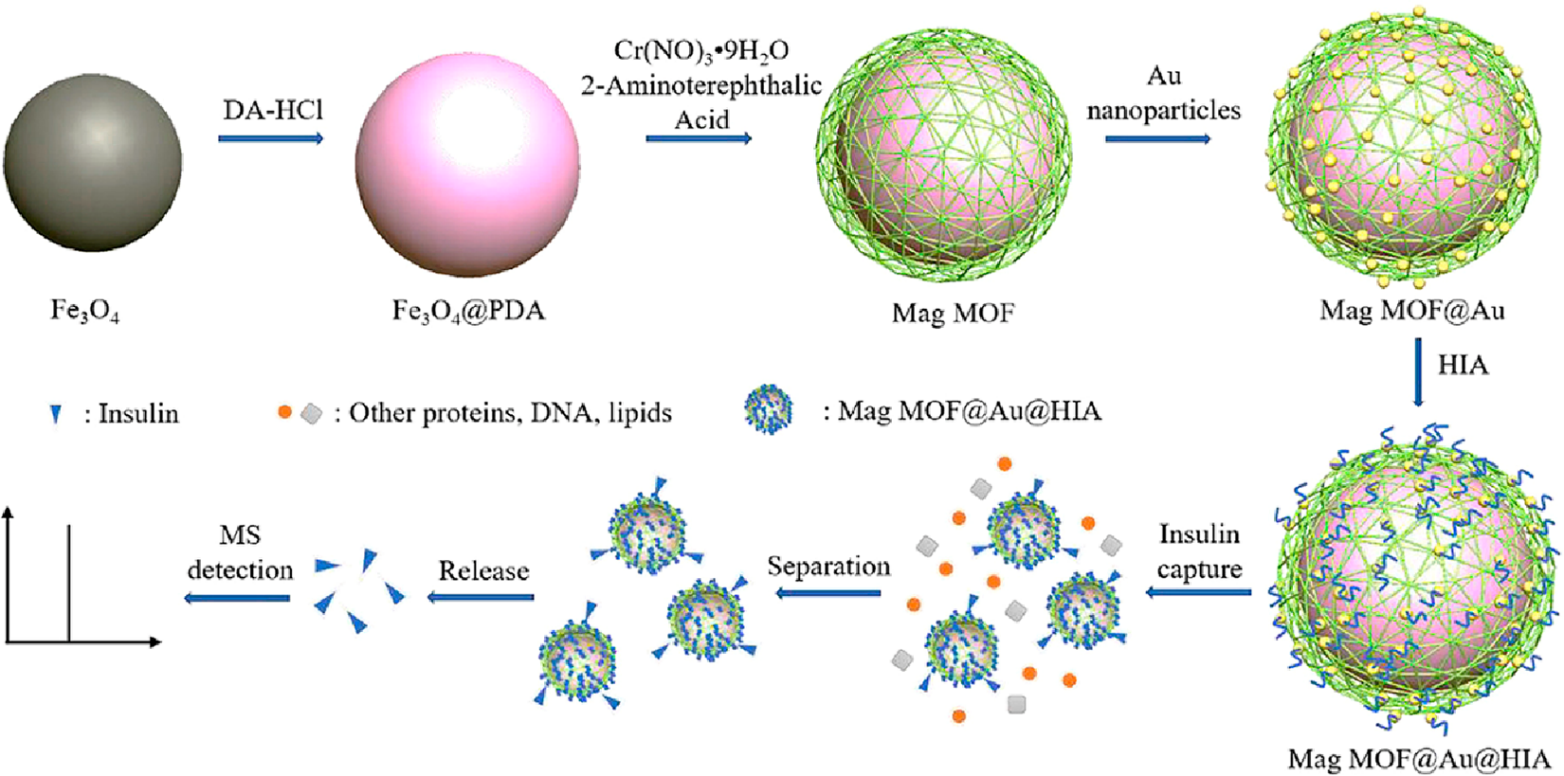
Schematic representation of the synthesis of Mag MOF@Au@HIA and workflow for the detection of insulin. Insulin binds the aptamers, and then, the particles are magnetically separated from the blood complex mixture. Upon release, insulin is detected by mass spectrometry. Adapted with permission from ref (90). Copyright 2019 Elsevier.

Exploring lanthanide fluorescence, Wang and co-workers developed a dual-detection MOF-based sensor for insulin.^89^ This sensor is a two-component system composed of a gadolinium/terphenyl-3,4″,5-tricarboxylic acid MOF, [Gd(L)(H_2_O)(DMF)]·DMF, where H_3_L stands for terphenyl-3,4″,5-tricarboxylic acid, and a fluorescence-labeled insulin aptamer (FAM-P). Upon contact with insulin, which binds the aptamer through hydrogen bonds, quenching of FAM-P occurs, a phenomenon potentiated by electrostatic interactions with the MOF.^89^ Insulin concentrations up to 1.8 × 10^3^ nM could be detected in a linear range (with 1.2 nM as the LOD), with the aptamer conferring high selectivity over other proteins such as recombination human growth hormone. Next, FeNPs coated with polydopamine were encapsulated into MIL-101(Cr) through the hydrothermal synthesis of the MOF itself. The MOF surface was then coated with AuNPs, and finally, an insulin aptamer (HIA) was grafted through Au–S bonds, yielding Mag (magnetic) MOF@Au@HIA (Figure 11).^90^ The MOF surface conferred an exceptional number of HIA functionalization spots, resulting in excellent insulin capture. The magnetic properties showed good responsiveness and allowed fast recovery of the particles following insulin incubation. Insulin detection was performed by MALDI-TOF MS analysis, with a detection range of 0.9 to 17 nM (LOD of 0.17 or 0.34 nM, for *in vitro* and in human serum, respectively). Despite requiring thermal denaturation of the aptamer prior to sample measurement, the system showed specificity for insulin over other serum proteins, such as albumin or IgG.

**Figure 12:**
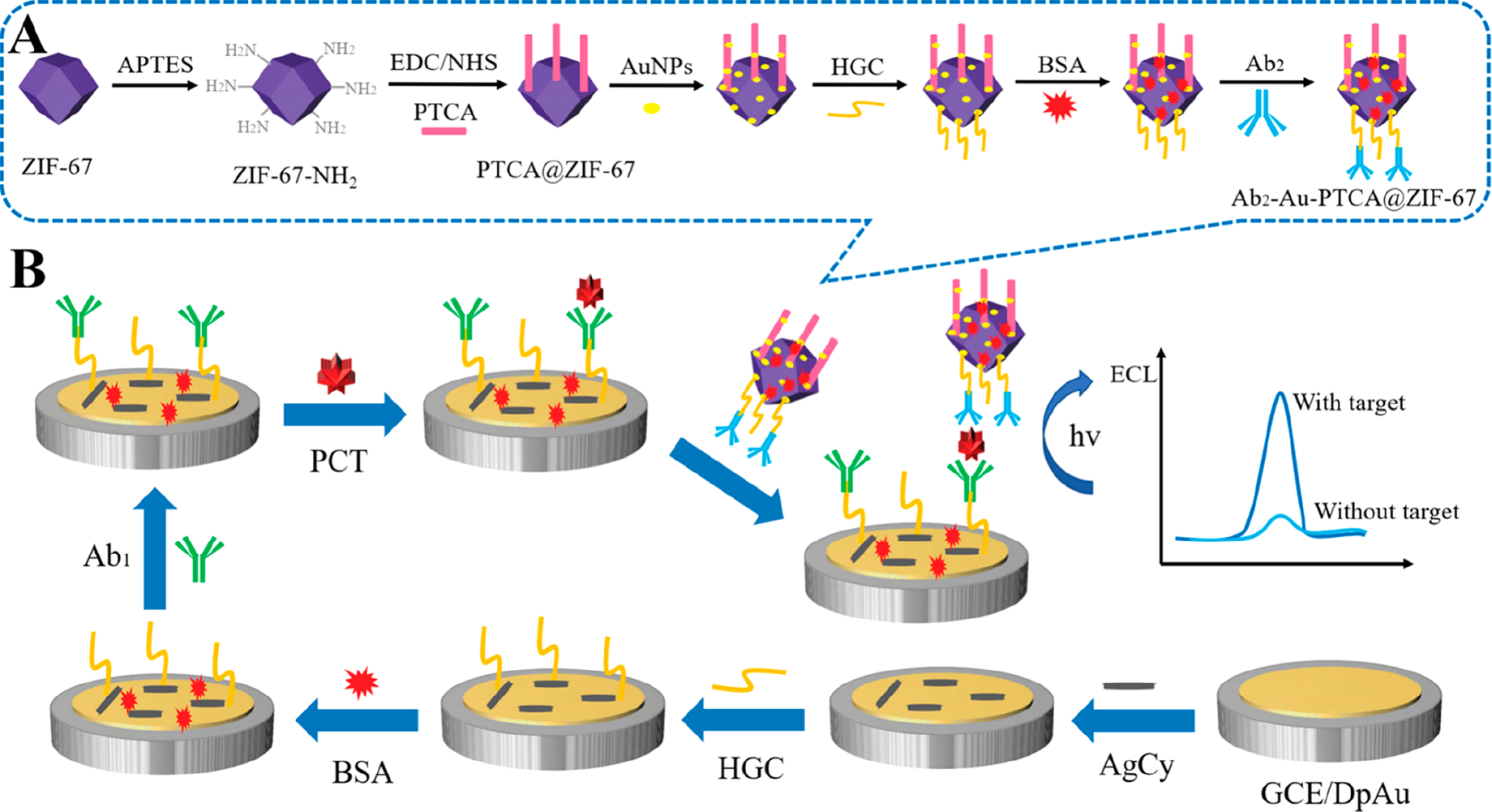
Schematic representation of the synthesis and workflow of a sensor for procalcitonin. (A) Preparation step of Ab_2_-Au-PTCA@ZIF-67 bioconjugates and (B) biosensor architecture. Adapted with permission from ref (91). Copyright 2020 American Chemical Society.

Wei and co-workers extended their biosensor portfolio by developing two similar GCE-anchored sandwich-type immunosensors for ECL detection of procalcitonin.^49a,54,56,57,61,87,91^ One is a double-quenching “signal on–off” sensor, with the first layer being composed of MIL-101(Al):Ru-PEI-Au-Ab_1_:MIL-101(Al), MIL-101(Al) encapsulating [Ru(bpy)_3_]^2+^, and subsequently functionalized with the ECL coreactant polyethylamine, PEI, and AuNPs to improve electron transfer efficiency for signal transduction. The second layer is composed of Fe_3_O_4_@PDA-Cu_*x*_O-Ab_2_ (polydopamine, PDA, and copper that provides a double quenching effect over [Ru(bpy)_3_]^2+^, which is “turned-off” upon procalcitonin binding).^92^ The other sensor consists of a layer of Au-AgCys-HGC (AgCys, silver cysteine particles; HGC, a low-cost peptide to bind an antibody against procalcitonin) and a layer of Au-PTCA@ZIF-67, with S_2_O_8_^2–^ as ECL coreactant (Figure 12). Here, the cobalt-based ZIF-67 MOF (isotypical to ZIF-8) provides two important advantages: high 3,4,9,10-perylenetetracarboxylic acid (PTCA) encapsulation and the incorporation of cobalt (a catalytic active metal site). PTCA is a powerful ECL donor, although it requires enhancers for a measurable signal detection. Such enhancement is achieved by cobalt, which acts as coreaction accelerator catalyzing S_2_O_8_^2–^ to generate abundant Co^3+^ and sulfate radical anions (SO_4_^•–^). Together with the increased antibody sensitivity conferred by the HGC peptide, cobalt and AgCys confer a “triple PTCA ECL signal amplification” capacity to this sensor.^91^ Both sensors perform well, with high selectivity for procalcitonin (over, for example, albumin or Aβ), stability, reproducibility, and performance with human serum samples (i.e., high recovery rates comparable to currently used ELISA kits). The “triple amplification” sensor is much more sensitive with a linear detection range of 6.9 × 10^–7^ to 6.9 nM and 2.53 × 10^–7^nM detection limit, against 3.5 × 10^–5^ to 6.9 nM and 1.24 × 10^–5^ nM LOD of the “signal on–off sensor”.^90,91^ Nevertheless, both are suitable to detect procalcitonin for potential MTC diagnosis, as the hormone levels in healthy individuals are below 6.9 × 10^–3^ nM.^26^

To achieve prolactin sensing, an MOF based on praseodymium and 5-aminoisophtalic acid (AIP) and 1,2-phenylenediamine (Phen) was prepared: [Pr(AIP)(Phen)Cl_2_(DMF)_2_(H_2_O)_2_] (Pr-MOF).^93^ Nanofibers were isolated with luminescence conferred by orbital transitions within the π-conjugated aromatic skeleton of the framework. Unlike most sensors that use an antibody or aptamer as recognition elements, prolactin interacts directly with the lone electron pair of the amine group composing the framework, resulting in metal-to-ligand charge transfer, as well as covalent bonding between the prolactin active site and the aromatic chromophore of Pr-MOF. This interaction results in a fluorescent signal that is proportional to prolactin concentrations, offering a linear range of detection up to 8 nM (LOD of 0.01 nM).^93,94^ Pr-MOF nanofiber prolactin detection is highly selective (over other hormones, such as thyroid stimulating hormone and luteinizing hormone) and presents good applicability to human serum samples (recovery rates around 100%), and good repeatability and reproducibility. Additionally, it offers a better reading range, lower production cost, and less time for sample measurement than other methods such as ELISA.^93^

### Autosomal Dominant Hereditary Systemic Amyloidosis Caused by Lysozyme and Apolipoprotein IV

**Figure 13:**
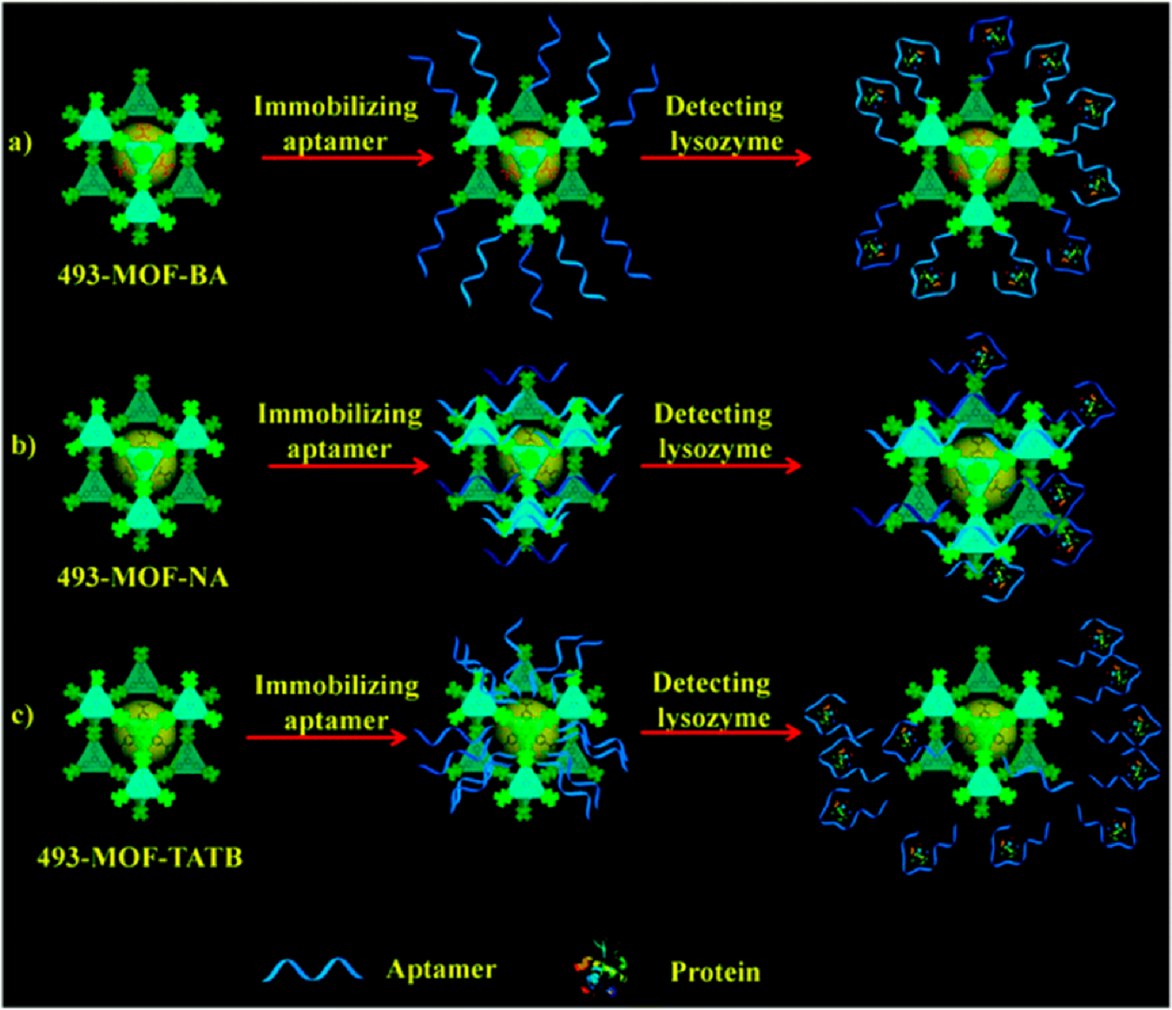
Schematic representation of the different binding modes of aptamers to 493-MOFs for lysozyme recognition: (a) 493-MOF-BA, (b) 493-MOF-NA, and (c) 493-MOF-TATB. Adapted with permission from ref (95). Copyright 2017 Royal Society of Chemistry.

Two MOF-based sensors were designed for the detection of autosomal dominant hereditary systemic amyloidosis caused by point mutations in lysozyme and apolipoprotein IV. For lysozyme targeting, Liu and co-workers designed an aptasensor based on a zirconium MOF formulated as [Zr_6_O_4_(OH)_4_(TATB)], where H_3_TATB stands for 4,4′,4″-*s*-triazine-2,4,6-triyltribenzoic acid and coined 493-MOF-BA.^95^ Initially, the authors modulated the MOF pore size by employing three organic linkers: benzoic acid, nicotinic acid, and tetrahydrofuran-2-carboxylic acid, yielding 493-MOF-BA, 493-MOF-NA, and 493-MOF-TATB, respectively. These MOFs display good water stability, which is a key factor for the analysis of biological fluids. The first two are isotypical, constructed from 3-fold interpenetrated frameworks, while 493-MOF-TATB has a noninterpenetrated porous network. The distinct ligands and porosity influenced the binding recognition mode of the lysozyme aptamer (Figure 13). For 493-MOF-BA, it binds through a phosphate–Zr interaction, adopting a perpendicular orientation to the framework (Figure 13a). In 493-MOF-NA, a stronger bond is established with nitrogen atoms of the pyridine rings of nicotinic acid, resulting in the aptamer being parallel to the framework (Figure 13b). Finally, aptamer binding was the weakest in 493-MOF-TATB due to the smaller pore sizes (Figure 13c).^95^ Overall, the higher aptamer availability made 493-MOF-BA the best for lysozyme detection. Briefly, the sensor consists of a gold surface coated with a thin layer of 493-MOF-BA, to which the aptamer binds. Lysozyme binding blocks basal electron transfer being dependent on the concentration, with a linear detection range from 3.4 × 10^–4^ to 6.8 × 10^–2^ nM (LOD of 2.4 × 10^–4^ nM). 493-MOF-BA exhibits good selectivity (over other circulating proteins such as albumin, thrombin, and IgG), stability, and reproducibility, while performing well with human serum samples (recovery rate over 94%).

ApoA4 specific detection was attained using a ZIF-8-based immunosensor,^91^ which employs a GCE surface upon which a nitrogen-doped graphene (N-Gr) combined with ZIF-8 is layered providing a vast specific surface area and excellent electrical conductivity. The material is then functionalized with 4-aminobenzoic acid (ABA) to which ApoA4 binds covalently. Subsequently, a biotinylated anti-apoA4 antibody binds the target protein. The electrochemical redox signal is generated by the action of labeled streptavidin with HRP that binds the biotin from the antibody and acts on the substrates *o*-phenylenediamine and hydrogen peroxide. This MOF-based sensor performed slightly better than the currently used ELISA detection method for ApoA4, both *in vitro* and with human samples. The ApoA4 linear concentration detection range goes from 3.2 × 10^–3^ to 6.6 nM, with an LOD of 1.8 × 10^–3^ nM. Adding to good reproducibility, stability, and selectivity (including against other apolipoproteins, such as ApoA1 or ApoC3), this sensor offers the possibility of being applied directly to a human sample with no prior treatment.

The characteristics of the sensors fabricated for human amyloidosis other than Alzheimer’s disease are presented in Table 2.

## Final Remarks

Human amyloid diseases, of which Alzheimer’s and Parkinson’s diseases are the most well-known examples, are conditions that carry significant costs to the individual patient and to society in general. In these diseases, biological disruption can start to manifest as much as a decade before they become severe enough for an upfront symptomatic diagnosis. While the search for disease-modifying treatments continues, it is vital that tools capable of early detection continue to evolve to a point where they become a standard practice among medical institutions around the world. Benefits of early diagnosis and treatment are highly important since most of the current available treatments for these diseases have a much higher chance of working on an early diagnosis basis. Consequently, there is great demand to develop a form of diagnosis for these diseases that does not require the vast expenses and difficulties that are currently needed with CSF and PET biomarkers. The latter is a very specialized imaging tool that is contraindicated in asymptomatic individuals because it is potentially more harmful than beneficial.^97^

In this context, MOF materials are an emerging component for amyloid biosensors. Although not limited to this, most of the reported MOF-based amyloid biosensors in the present review target AD’s. This discrepancy may be explained by the ease of obtaining each *in vitro* target analyte—Aβ peptides are supplied by several companies, while, for example, α-synuclein requires more laborious recombinant protein production techniques. All the biosensors reviewed here are designed to carry out target monitoring by noninvasive collection of human fluids, such as CSF or plasma. Not surprisingly, immunosensors and aptosensors perform consistently better, sensitivity wise. MOFs have been gaining a leading role in the fabrication of these devices due to their versatility. They can encapsulate high loads of emission or receptor probes, protecting the guest from solvent, and simultaneously can be doped by metal nanoparticles, such as AuNPs, for better signal transduction and immobilization of antibodies or aptamers.

### Do MOF-Based Sensors Meet Application Requirements for Amyloid Targets?

Several ingenious devices were fabricated that can detect femtomolar concentrations of the amyloid target, which agrees with the overall detection limit reached for other biomolecules, and thus, further significant improvements are not expected. They also outperform sensors built without MOFs. For example, for Alzheimer’s disease Aβ peptide detection, the electrochemiluminescence dual-MOF Ru(bpy)_3_^2+^-NH_2_-UiO-66-Ab_1_/Ab_2_-MIL-101@Au-MoS_2_ QDs/TPA sensor, with a detection range of 10 fg mL^–1^ to 50 ng mL^–1^ and an LOD of 3.32 fg mL^–1^,^56^ performed better than a silver nanocluster/titanium nanomaterial hybrid-based sensor (detection range of 50 fg mL^–1^ to 50 ng mL^–1^ and limit of 32 fg mL^–1^);^98^ the fluorescent Ru-MIL-101(Al)-Apt-AuNPs/RecJF sensor (detection range of 1 pM to 10 nM and limit of 0.3 pM)^64^ is superior to a quantum dot-based sensor (detection range of 5 to 8 nM and limit of 0.2 nM);^99^ and the electrochemical dual-signal Cu-Al_2_O_3_-*g*-C_3_N_4_-Pd Ab_1_/UiO-66@PANI-MB Ab_2_ sensor (detection range of 10 fg mL^–1^ to 100 ng mL^–1^ and limit of 3.3 fg mL^–1^)^54^ outperforms a sensor based on the phenolic pigment curcumin (detection range of 1 pM to 5 nM and limit of 1 pM).^100^ For Parkinson’s disease, the MOF-based sensors for α-synuclein oligomer detection (range of around 1 fM to 0.5 pM and limit of around 0.4 fM)^20^ proved to perform better than an enzyme/gold-based sensor (detection range of 60 pM to 150 nM and limit of 10 pM).^101^ Furthermore, there is a consensus about blood biomarkers and the way these would facilitate not only diagnosis but also research outside of the few urban academic centers that have the capacity to collect currently used CSF-extracted or PET biomarkers. MOF-based sensors have already achieved this stage and are thus pushing these materials beyond the lab bench. When compared with ELISA for clinical diagnosis, multiple MOFs biosensors herein reported showed, at least, a similar analytical performance.^20,53,56,90,91,93,96^ These sensors reduce, however, the overall complexity and length of currently used protocols, making them more appealing for a clinical setting (measuring times can be as low as 15 min).^61^ Interestingly, the use of MOFs in the field of human amyloids can go beyond the sensing purpose. Although clearly in its infancy, a few research groups have explored the versatility of these materials to inhibit amyloid aggregation^79,82,102^ or develop *in vitro* tools as an alternative to the widely used amyloid dye thioflavin-T to follow aggregation, with particularly good results in detecting the most cell damaging early oligomeric intermediates.^73^

### Sensors for Alzheimer’s Disease—What Should We Measure?

One of the most distinct features of Alzheimer’s disease is the early deposition of amyloid plaques. The main components of these plaques are the Aβ peptides. Aβ_1–42_ is less soluble than Aβ_1–40_ and is more prone to form amyloid aggregates. Aβ_1–40_ levels in the CSF, the predominant of the several Aβ isoforms (nearly 10 times more abundant than Aβ_1–42_), remain unchanged during the progression of the disease. Thus, sensors should be highly selective toward Aβ_1–42_ (the reactive species) against the dominant Aβ_1–40_ isoform. Ideally, as the production of Aβ varies from individual to individual, dual-probe (Aβ_1–42_/Aβ_1–40_) sensors should be fabricated so that the Aβ_1–40_ concentration may be used to normalize individual basal Aβ production. This is important because Aβ basal production varies from individual to individual and thus Aβ_1–42_, making the determination of Aβ_1–42_/Aβ_1–40_ more reliable than that of Aβ_1–42_ alone.^15a^ These distinct features of the Aβ isoforms are frequently overlooked in the sensors described in the literature. The devices are rarely tested with the two species Aβ_1–42_ and Aβ_1–40_, and in some cases, they are developed for the Aβ_1–40_ isoform or for an unspecified Aβ peptide.

On the other hand, there is an intense debate whether the amyloid pathway is causative or a side effect of the disease. This is a key question to define the most plausible therapeutic strategy: should we go after Aβ oligomers or invest in other potential causes such as inflammation or immune dysfunction? Thus, the ability to measure the levels of Aβ oligomers selectively in body fluids is relevant and was the aim of some of the fabricated sensors. Even more crucial is to clarify whether there is a correlation between local accumulation of Aβ oligomers and functional or structural brain damage. MOFs’ application in amyloid imaging is virtually unexplored. Metal clusters on MOFs and their high capacity to encapsulate imaging guests render them viable candidates as contrast agents for imaging techniques such as magnetic resonance imaging and computed tomography. The ability to modulate particle size and to produce nanoscale particles, together with the ability to attach recognition motifs such as antibodies to the particle’s surface, may soon selectively provide contrast agents for Aβ oligomers. Nowadays, radioligands are used for PET imaging that do not distinguish the size of the fibrils. Although the application of MOFs in medicine is in its early stages, there is a huge discrepancy between the research effort dedicated to the synthesis of MOF for targeted tumor imaging when compared to targeting amyloid species. Hopefully, this gap will be shortened in the near future.

### Future Prospects—What Should Be Done?

MOFs are nowadays well-established materials in the biosensing research area, being important components in the design of devices for the detection of a wide array of diseases. Much can still be done in this area of research, and aspects such as with the collection of viable samples remain the most obvious critical step to overcome before MOF-based sensors, or any other sensor developed for *in situ* applications, can be put to practical use. New MOF biosensors should meet the healthcare needs of people, saving public medical resources by extending their biosensing capabilities to real-life situations. Unmistakably we believe that in the upcoming years the continuous development of MOF sensors, as summarized in this Review, might lead to simple and effective commercial diagnostic tools.

## Abbreviations

2Im: 2-methyl imidazole
493-MOF-BA: [Zr_6_O_4_(OH)_4_(TATB)]
α-syn: α-synuclein
Ab: antibody
ABA: 4-aminobenzoic acid
ABEI: *N*-(aminobutyl)-*N*-(ethylisoluminol)
AD: Alzheimer’s disease
AgCys: silver cysteine particles
AIP: 5-aminoisophtalic acid
ApoA4: apolipoprotein A4
Apt: aptamer
APTMS: 3-aminopropyl-trimethoxysilane
AuNFs: gold nanoflowers
Aβ: amyloid-β
CD: cyclodextrin
CSF: cerebrospinal fluid
EC: electrochemical
ECL: electrochemiluminescent
ECL-RET: electrochemiluminescence–resonance energy transfer
EDC: 1-ethyl-3-(3-(dimethylamino)propyl)carbodiimide
Er-MOF: [Er(L)(DMF)_1.27_]
FAM-P: fluorescence-labeled insulin aptamer
FeNPs: iron nanoparticles
GCE: glass carbon electrode
H_2_bdc: 1,4-benzenedicarboxylic acid
H_2_bdc-NH_2_: 2-amino-1,4-benzenedicarboxylic acid)
H_2_bpdc: 4,4′-biphenyldicarboxylic acid
H_2_TCPP: tetrakis(4-carboxyphenyl)porphyrin
H_3_BTC: 1,3,5-benzenetricarboxylic acid
H_3_HTB: 4,4′,4″-(1,3,3a1,4,6,7,9-heptaazaphenalene-2,5,8-triyl)tribenzoic acid
H_3_L: terphenyl-3,4″,5-tricarboxylic acid
H_3_TATB: 4,4′,4″-*s*-triazine-2,4,6-triyltribenzoic acid
H_3_tatb: triazine-1,3,5-tribenzoic acid)
H_4_TBAPy: 1,3,6,8-tetrakis(*p*-benzoate)pyrene)
HIA: insulin aptamer
HKUST-1: [Cu_3_(BTC)_2_(H_2_O)_3_]
HRP: horseradish peroxidase
Im: imidazole
ITO: indium tin oxide
LIDA: localized insulin-derived amyloidosis
LPFFD: H-Leu-Pro-Phe-Phe-Asp-OH trifluoroacetate salt
MIL-100: [Fe_3_F(H_2_O)_2_O(btc)_2_]·28.5H_2_O
MIL-101: [Fe_3_OH(H_2_O)_2_O(bdc)_3_]
MIL-101_NH_2_: [Fe_3_OH(H_2_O)_2_O(bdc-NH_2_)_3_]
MIL-53: [Fe(OH)(bdc)]·H_2_O
MIL-53-NH_2_: [Fe(OH)(bdc-NH_2_)]·H_2_O
MIL-88A: [Fe_3_O(fumarate)_3_]_*n*_
MIL-89: [Fe_6_O_2_(fumarate)_6_]
MOF-5: [Zn_4_O(bdc)_3_]
MTC: medullary thyroid cancer
N-Gr: nitrogen-doped graphene
NFT: neurofibrillary tangle
NHS: hydroxysuccinimide
NiFe-MOF: Ni_3_[Fe(CN)_6_]_2_·10H_2_O)
NIR: near-infrared
NU-1000: [Zr_6_(μ^3^-OH)_8_(OH)_8_(TBAPy)_2_]
PANI: polyaniline
PCN: porous coordination network
PCN-222: [Zr_6_(μ^3^-O)_8_(OH)_8_(TCPP)_2_]
PCN-224: [Zr_15_(TCPP)_3_(μ^3^-OH)_16_(OH)_20_(H_2_O)_4_]
PCN-888: ([Al_3_O(OH)(H_2_O)_2_(HTB)]
PD: Parkinson’s disease
PDA: polydopamine
PEI: polyethylamine
Phen: 1,2-phenylenediamine
Pr-MOF: [Pr(AIP)(Phen)Cl_2_(DMF)_2_(H_2_O)_2_]
PTCA: 3,4,9,10-perylenetetracarboxylic acid
Ru(bpy)_3_^2+^-Zn-oxalate-MOF: [Ru(bpy)_3_][Zn_2_(C_2_O_4_)_3_]
siRNA: small interfering ribonucleic acid
Tb-mesoMOF: [Tb_16_(tatb)_16_(DEA)_24_]·91(DEA)·108(H_2_O)
THS: triple helix switch
ThT: benzothiazole thioflavin-T
TPA: tripropylamine
UiO-66: [Zr_6_O_4_(bdc)_2_]
UiO-67: ([Zr_6_O_4_(OH)_4_(bpdc)_6_]
ZIF-67: [M(2Im)_2_]) with M = Co/Ni
ZIF-8: [Zn(Im)_2_]

## Vocabulary

amyloid: aggregates, mainly formed by proteins, associated with several diseases such as Alzheimer’s and Parkinson diseases. Amyloids may arise from several proteins but share common characteristics such as a fibrillar morphology, high content in β-sheet secondary motif and the ability to be stained by specific dies.
Amyloid β-peptide: short peptide (36 to 43 residues) derived from the sequential cleavage of the β-Amyloid Precursor Protein. Amyloid β-peptide accumulation in the brain is associated (but not necessarily as the cause of) with Alzheimer’s disease. Sensors are being developed for detection of amyloid β-peptide species in the cerebrospinal fluid.
Metal–Organic Frameworks (MOFs): class of ordered porous solids consisting of metal ions or clusters coordinated to organic ligands. Researchers began to explore the potential of MOFs in several applications, namely in the development of amyloid sensors, due to their tunable composition and pore geometry.
Antibody (immunosensor): protein produced by the body’s immune system that recognizes a unique molecule, called the antigen. Sensors that use antibodies as recognition motifs are immunosensors.
Aptamer: short oligonucleotides that selectivity bind to a specific molecule, including a protein. Can be used as biorecognition motif to fabricate a sensor. Aptamers are as much as 100 times smaller than antibodies but are prone to fast degradation in biological media.
